# Unraveling the structure and function of a novel SegC protein interacting with the SegAB chromosome segregation complex in Archaea

**DOI:** 10.1093/nar/gkae660

**Published:** 2024-07-30

**Authors:** Min-Guan Lin, Cheng-Yi Yen, Yo-You Shen, Yu-Sung Huang, Irene W Ng, Daniela Barillà, Yuh-Ju Sun, Chwan-Deng Hsiao

**Affiliations:** Institute of Molecular Biology, Academia Sinica, Taipei 115, Taiwan; Institute of Molecular Biology, Academia Sinica, Taipei 115, Taiwan; Institute of Molecular Biology, Academia Sinica, Taipei 115, Taiwan; Institute of Bioinformatics and Structural Biology, National Tsing Hua University, Hsinchu 300, Taiwan; Department of Biology, University of York, Wentworth Way, YorkYO10 5DD, UK; Department of Biology, University of York, Wentworth Way, YorkYO10 5DD, UK; Institute of Bioinformatics and Structural Biology, National Tsing Hua University, Hsinchu 300, Taiwan; Institute of Molecular Biology, Academia Sinica, Taipei 115, Taiwan

## Abstract

Genome segregation is a fundamental process that preserves the genetic integrity of all organisms, but the mechanisms driving genome segregation in archaea remain enigmatic. This study delved into the unknown function of SegC (SSO0033), a novel protein thought to be involved in chromosome segregation in archaea. Using fluorescence polarization DNA binding assays, we discovered the ability of SegC to bind DNA without any sequence preference. Furthermore, we determined the crystal structure of SegC at 2.8 Å resolution, revealing the multimeric configuration and forming a large positively charged surface that can bind DNA. SegC has a tertiary structure folding similar to those of the ThDP-binding fold superfamily, but SegC shares only 5–15% sequence identity with those proteins. Unexpectedly, we found that SegC has nucleotide triphosphatase (NTPase) activity. We also determined the SegC-ADP complex structure, identifying the NTP binding pocket and relative SegC residues involved in the interaction. Interestingly, images from negative-stain electron microscopy revealed that SegC forms filamentous structures in the presence of DNA and NTPs. Further, more uniform and larger SegC-filaments are observed, when SegA-ATP was added. Notably, the introduction of SegB disrupts these oligomers, with ATP being essential for regulating filament formation. These findings provide insights into the functional and structural role of SegC in archaeal chromosome segregation.

## Graphical Abstract

**Graphical Abstract:**
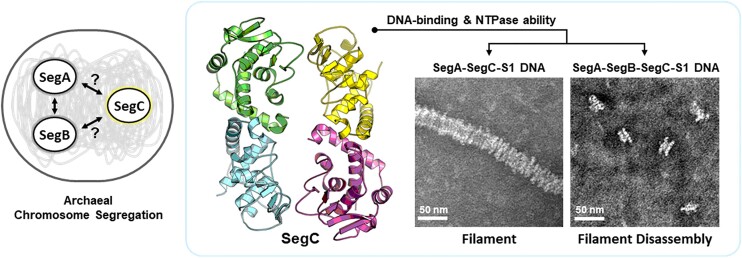

## Introduction

Chromosome replication and segregation are vital processes in all living organisms, ensuring the preservation of genetic material. The mechanisms of genome segregation have been extensively studied in both eukaryotic and prokaryotic cells. In eukaryotes, mitotic chromatids are well defined and pulled apart by the mitotic spindle and motor proteins during chromosome segregation (1). Bacterial DNA partitioning systems consist of three components—an NTPase ParA, a centromere-binding protein (CBP) ParB, and centromere-like DNA site *parS*—with several DNA segregation mechanisms having been proposed (2–4).

However, our knowledge of the chromosome segregation mechanism is more limited for Archaea, the third kingdom of life that is widely believed to include the ancestors of eukaryotes (5,6). Although archaea have small circular genomes like those of bacteria, they replicate and organize their genomes in diverse and unique ways (7,8). In recent years, metagenomics studies have led to the discovery of novel archaeal lineages, such as the Asgard superphylum that includes the closest archaeal relatives of eukaryotes (9,10). Currently, the archaea domain comprises three superphyla: TACK (Thaumarchaeota, Aigarchaeota, Crenarchaeota, and Korarchaeota) (11), DPANN (Diapherotrites, Parvarchaeota, Aenigmarchaeota, Nanohaloarchaeota, and Nanoarchaeota) (12) and Asgard superphylum, in addition to the Euryarchaeota phylum. Previous studies have shown that some archaea, including the euryarchaeal genus Haloferax, have multiple copies of their genome organized into distinct nucleus-like compartments (or nucleoids) in a single cell (13). In contrast, crenarchaeal Sulfolobales and the thaumarchaeon *Nitrosopumilus* inherit a single-copy chromosome, rendering cell-cycle regulation critical for survival (7,13). Sulfolobales undergo an ordered cell cycle with distinct phases of DNA replication and segregation, resembling the cell cycle observed in many eukaryotes (14–16).

Recent investigations have shed light on the molecular mechanism underlying archaeal chromosome segregation, specifically the SegAB system found in *Saccharolobus solfataricus* (previously *Sulfolobus solfataricus*) (17,18). *In vivo* analyses have shown that increased expression of either the *segA* or *segB* gene results in a high percentage of anucleate cells, indicating that SegAB acts as a primary mediator of chromosome segregation in *S. solfataricus* (17). Sequence analysis and structural data have revealed that SegA is a Walker-type ATPase that resembles bacterial ParA orthologs. SegB is an archaea-specific centromere-binding protein (CBP) that specifically recognizes palindromic centromere-like sites, including site 1 (S1) located upstream of the *segA* start codon and site 2 (S2) centred at position -59 located upstream of the same start codon (17,18). Cooperation between the SegA and SegB proteins enables DNA packaging and organization for archaeal chromosome segregation. Moreover, studies have demonstrated that both the *segA* and *segB* genes are expressed in early S phase and are later downregulated by the cell cycle regulator aCcr1 in the D and G1 phases (19,20). These findings indicate that the SegA and SegB proteins might begin to organize the chromosome during the replication stage.

Given that SegA is an archaeal ortholog of bacterial ParA, it might deploy a mechanism similar to that proposed for ParA in segregating the two replicated chromosomes. However, ParA-mediated chromosome segregation is a dynamic process regulated by cycles of ATP binding and hydrolysis, which promote the movement of the ParB-coated *parS* region of the chromosomes through a diffusion-ratchet mechanism (21–23). Upon ATP hydrolysis, ParA-ADP dissociates from the nucleoid and the ParA-void area triggers the large ParB-bound *parS* complex to chase and move to another ParA-ATP dimer bound to the nucleoid (23). Consequently, the newly replicated origin region near the *parS* will be carried by ParB-*parS* complex and move along the ParA concentration gradient. The mechanism of chromosome segregation relies on the crucial ATP hydrolysis ability of ParA, which regulates the association and dissociation of ParA from DNA (21). For bacterial ParA proteins the assembly of an ATP-bound sandwich dimer is an obligate step to build up substantial protein surface that will enable DNA binding (21).

In contrast to ParA, SegA exhibits a unique forward-backward functional dimer conformation, resulting in a DNA binding mode that is not restricted by ATP (18). Thus, SegA does not necessitate ATP binding and hydrolysis for association to and release from DNA. In view of these differences, SegA might not adopt a bacterial ParA-like mechanism to separate chromosomes or, alternatively, other auxiliary factors might be involved in archaeal chromosome segregation. We now have some understanding of chromosome segregation in the Archaea, but some elements of the process remain unclear, including some contributory factors. For instance, a gene of unknown function localized ∼100 base pairs (bp) upstream of the *segA* gene, initially annotated as *sso0033* (later SSO_RS160), may play a role in chromosome segregation (17). DNase I footprint results showed that the window of protection generated by SegB on the DNA covered the start site of the upstream gene *sso0033*, suggesting a potential functional link between *sso0033* and *segAB* cassette (17).

To elucidate the full picture of archaeal chromosome segregation, we investigated the *sso0033* gene of unknown function harbored by the *S. solfataricus* chromosome. We examined the biochemical function of the SSO0033 protein and determined its crystal structure. The crystal structure revealed that SSO0033 adopts a dimeric conformation as a basic building block that can form various multimeric structures. Surprisingly, we found that SSO0033 exhibits nucleotide triphosphatase (NTPase) activity and assembles into filaments in the presence of nucleoside triphosphates (NTPs) and DNA. Furthermore, negative-stain electron microscopy images of an SSO0033-SegA-DNA ensemble showed long and highly organized filament formations in the presence of ATP, which collapsed and dissolved upon addition of SegB. In view of our findings that indicate that SSO0033 interacts with the SegAB complex, we have named this protein SegC. Our results provide insights into the role of SegC in the mechanism of chromosome segregation in the archaeon *S. solfataricus*.

## Materials and methods

### Protein expression and purification

SegA, SegB and mutant variants were expressed and purified as described previously (17,18). The *segC* gene and the *segC*_1–155_ mutant variant were cloned into pET21b (+) (Novagen) vector with a C-terminal His_6_ tag for protein expression in *Escherichia coli* BL21 (DE3). Transformed *E. coli* cells were grown on an LB-agar plate containing 100 μg/ml ampicillin at 37°C for 16 h. Colonies were scraped off and transferred into 200 ml LB medium for cell proliferation at 37°C for 2 h. Saturated SegC cultures in LB medium were diluted 50-fold in 2 L of fresh medium and cultivated at 37°C with shaking at 180 rpm. Then, 1 mM isopropyl-β-d-1-thiogalactopyranoside (IPTG) was added when the optical density (OD_600_ nm) reached 0.8. The SegC overproduction culture was then incubated at 30°C for 3 h. Cells were harvested by centrifugation at 4500 *g* for 30 min at 4°C and stored at –80°C.

Cell pellets were suspended in lysis buffer containing 20 mM HEPES–NaOH pH 7.5 and 1 M NaCl. The suspended cells were disrupted by microfluidizer, before being heated at 65°C for 15 min. To remove the debris, the cell lysate was centrifuged at 35 000 *g* for 30 min at 4°C. The supernatant was filtered through a membrane with a pore size of 0.22 μm, before loading the filtrate onto a HisTrap HP column (Cytiva) equilibrated with lysis buffer. After washing with 50 ml of lysis buffer, the target protein was eluted by applying an imidazole gradient from 0 to 1 M in the same buffer. All purified proteins were subjected to SDS-PAGE analysis.

### Cross-linking of SegC using bis(sulfosuccinimidyl) suberate (BS^3^) crosslinker

The cross-linking reaction was carried out in a 25 μl reaction mixture containing 80 μM SegC and BS^3^ crosslinker (Thermo Scientific™) at the indicated concentration in a buffer of HEPES–NaOH pH7.6, 100 mM NaCl and 5 mM MgCl_2_ at room temperature for 30 min. At the end of the reaction, the unreacted cross-linker was quenched by adding 2 μl of 675 mM Tris–HCl pH 8.0 (final 50 mM) to the mixture. The cross-linked proteins were resolved by 4–12% SDS-PAGE, and the bands were visualized by staining with Coomassie brilliant blue.

### DNA preparation

We procured a 24-bp non-specific DNA (nsDNA) (F: AGGGTGTTCCACGTGAAACAGGGA; R: TCCCTGTTTCACGTGGAACACCCT) containing a scrambled DNA sequence, as well as site-specific 21-bp DNA (F: ACGTAGAAGAGTCTAGACTGA; R: CAGTCTAGACTCTTCTACGTA) and 23-bp DNA (F: TACGTAGAAGAGTCTAGACTGAC; R: TCAGTCTAGACTCTTCTACGTAG) containing a site 1 (S1) sequence, respectively (27). Oligonucleotides were suspended at a 1:1 molar ratio of complementary DNA sequences in buffer containing 20 mM Tris–HCl pH 7.5, 100 mM NaCl and 2 mM MgCl_2_. After incubation at 95°C for 10 min, the solution was slowly cooled to room temperature, and the DNA substrate was stored at –20°C.

### Fluorescence polarization binding isotherms

Fluorescence polarization (FP) binding isotherms were conducted to assess the equilibrium DNA-binding properties of SegC and interactions among SegA, SegB and SegC. The DNA substrates used in the assays were fluorescently labeled at the 5′ end, enabling measurement of fluorescence polarization in the protein:DNA complex compared to unbound DNA. A two-fold serial dilution of SegC protein, starting from 20 μM, was prepared in a storage buffer containing 20 mM Tris–HCl pH 7.5, 100 mM NaCl, and 2 mM MgCl_2_. Subsequently, the protein samples were pre-incubated with 5 nM Cyanine-3 (Cy3)-labeled DNA at room temperature.

To determine the binding constants of SegA, SegB and SegC, SegC was labeled with Alexa488 green-fluorescent dye using an Alexa Fluor™ 488 Microscale Protein Labeling Kit (Invitrogen™). 10 μM SegA or SegB proteins with 2-fold serial dilution were mixed with 10 nM fluoresceinated SegC and incubated at room temperature for 30 min. The sample was incubated with buffer contained 20 mM HEPES–NaOH pH 7.3, 100 mM NaCl and 2 mM MgCl_2_.

The fluorescence polarization of the Cy3-labeled DNA or fluoresceinated SegC in the presence of the buffer alone represented the unbound state, respectively. Binding assays were performed by monitoring changes in fluorescence polarization using a Paradigm plate reader (Molecular Devices). The fluorescence polarization signal was measured at 595 nm with an excitation wavelength of 535 nm for Cy3-labeled DNA. The fluoresceinated SegC signal was determined at excitation wavelength of 485 nm and emission wavelength of 535 nm. The concentration of protein required to bind 50% of the Cy3-labeled DNA or fluoresceinated SegC was calculated, respectively, and the average of three independent experiments was determined, with error bars indicating the standard deviations.

### Electrophoretic mobility shift assay (EMSA)

A band-shift reaction was conducted in 20 μl containing 20 mM Tris–HCl pH 7.5, 100 mM NaCl, 2 mM MgCl_2_ and 10 nM 5′ end Cy3-labeled DNA substrate with various concentrations of SegC. The reactions were incubated at 37°C for 30 min, before adding native gel running dye (10 mM Tris–HCl pH 7.6 and 5% glycerol final concentration) directly to the samples. The complexes were separated through 4–12% TBE polyacrylamide gels for electrophoresis in 0.5× TBE buffer (45 mM Tris-borate pH 8.0 and 1 mM ethylene diamine tetraacetic acid (EDTA)) for 100 min at 70 V. Gels were immediately scanned for fluorescence signals using the Cy3 channel with a Typhoon FLA9000 system (GE Healthcare) to visualize DNA bands.

### Crystallization, data collection and structure determination

SegC buffer was gently exchanged to 20 mM Tris–HCl pH 7.5, 100 mM NaCl, and 2 mM MgCl_2_ via an Amicon® Ultra-0.5 10K centrifugal filter tube (Cytiva). Crystallization was performed manually using the hanging-drop vapor diffusion method at 20°C. Crystal plates were set up with 4 mg/ml SegC and a variety of commercial screens, using 1 μl protein sample mixed with 1 μl reservoir solution. The crystals were grown in 100 mM Tris–HCl pH 8.5 and 300 mM potassium thiocyanate. SegC crystals of maximum size were obtained in one week. Monomeric SegC_m_ crystals were grown in 20 mM calcium chloride, 100 mM sodium acetate pH 4.6 and 30% v/v MPD, and the crystals were observed after a couple of months. The SegC-ADP complex was prepared by soaking crystals within one minute in a solution containing 20 mM ATP, 20 mM Tris–HCl pH 7.5, 100 mM NaCl, and 2 mM MgCl_2_. Crystals were flash-cooled in liquid nitrogen at 100 K.

X-ray diffraction data for SegC and SegC_m_ crystals were collected from beamlines TPS 07A and 05A, National Synchrotron Radiation Research Center, Taiwan, respectively. The resulting dataset was processed using the HKL-2000 software (24). The phase of the SegC structure was determined by molecular replacement (MR) in *Phaser* (25) using the AlphaFold (26) model of SegC as a search model. The ATP soaked structure was solved as above but with the native SegC structure as the search model. However, initial *F*_o_ – *F*_c_ difference density maps revealed sufficient additional electron density to account for the presence of the ADP molecule. Structural refinement was performed in *PHENIX* (27), and adjustment of the structural model was performed in *COOT* (28). Detailed X-ray diffraction data and structural refinement statistics are summarized in Supplementary Table S1.

### NTPase assay

Steady-state NTPase activity assays were performed according to the malachite green method with some modification (29). 10 μM protein (SegA, SegB, SegC or SegC mutant) was incubated with or without 1 μM DNA and 1 mM NTP. The reaction was conducted in a buffer of 20 mM Tris–HCl pH 7.5, 100 mM NaCl and 2 mM MgCl_2_, with a final volume of 200 μl, at 37 °C for 1 h. The reaction was terminated by adding 200 μl 10% SDS, followed by addition of 200 μl of 1.25% ammonium molybdate in 6.5% H_2_SO_4_ and 200 μl of 9% ascorbic acid for coloring. The hydrolyzed phosphate product and molybdic acid form a complex that can be reduced upon encountering ascorbic acid to generate a deep blue color that is monitored at 660 nm. ATPase activity was determined under standard assay conditions. Three independent repeats for NTPase assays were conducted, with error bars representing standard deviations.

### Electron microscopy

All negative-stain electron microscopy experiments were performed in 20 mM Tris–HCl pH 7.5, 100 mM NaCl and 5 mM MgCl_2_. The proteins (SegA, SegB or SegC) and S1 DNA were mixed at 37 °C for 30 min. Then, 1 mM ATP, ADP or NTP was added to the mixture and placed on ice before grid preparation. SegC protein (20 μM) was prepared for the control images. All protein-DNA complex experiments were conducted using the 23-bp S1 DNA at a 10:1 (protein:DNA) molar ratio. For the SegC-S1 DNA complex, we mixed 20 μM SegC with 2 μM S1 DNA. For the SegC-S1 + NTP complex, 20 μM SegC was mixed with 2 μM S1 DNA and 1 mM NTP. For the [SegC-S1] – [SegA + ATP/ADP] complex, we mixed 20 μM SegA, 20 μM wild type (WT) SegC, 1 μM S1 DNA and 1 mM ATP or ADP. For the [SegC-S1] – [SegA + ATP] – SegB complex, 20 μM SegA, 20 μM SegB, 20 μM SegC, 1 μM S1 DNA and 1 mM ATP were mixed. For the [SegC-S1] – [SegB + ATP] complex, we mixed 20 μM SegA, 20 μM wild type (WT) SegC, 1 μM S1 DNA and 1 mM ATP. The samples were placed on a clean parafilm surface and then picked up onto a carbon-coated grid before being negatively stained with 1% uranyl acetate. After the grid had been air-dried for 1 day, images were captured using a Tecnai G2 Spirit TWIN (Thermo) electron microscope at a magnification of ×26 000 at 120 kV. Protein–DNA complex length and width were measured using ImageJ. Image frames were randomly selected from different grids.

## Results

### SegC shows a non-specific DNA binding activity

The *segC* (*sso0033*) gene encodes a 165-residue hypothetical protein of unknown function. This gene is located upstream of the *segAB* cassette that regulates chromosome segregation in the Archaea (17). The *segC* gene is only found in three genera of Sulfolobaceae (*Saccharolobus*, *Sulfolobus* and *Metallosphaera*), with 32–83% sequence identity at protein level (Supplementary Figure S1) (17). A previous DNaseI footprinting analysis indicated that SegB binds and protects the DNA region that controls the expression of the *segC* gene (17). Therefore, it is plausible that *segC* may be involved at some level in archaeal chromosome segregation.

**Figure 1.**
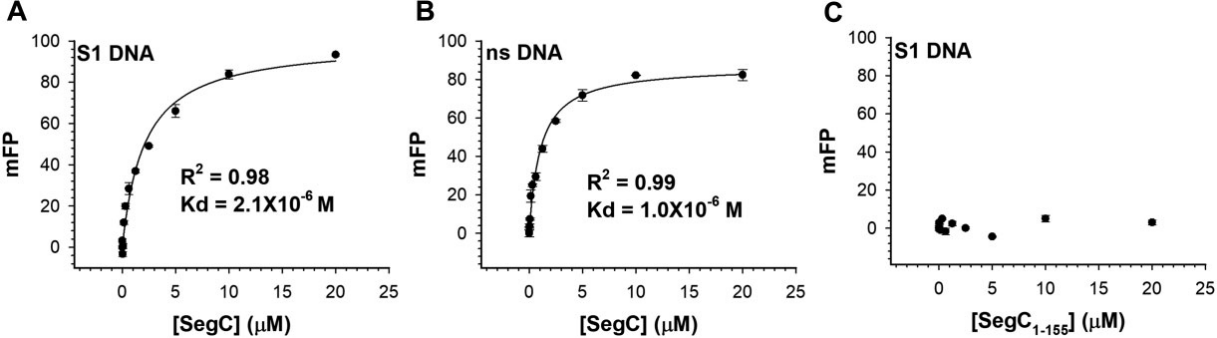
DNA-binding ability of SegC, as determined by fluorescence polarization (FP) binding isotherms. SegC incubated with (**A**) 21-bp site 1 (S1) double-stranded DNA (dsDNA) and (**B**) 23-bp non-specific dsDNA. (**C**) The SegC_1–155_ mutant incubated with 21-bp S1 dsDNA. All measurements are reported in triplicate and error bars represent the standard deviation of the mean. The solid lines represent fitting curves to the Michaelis–Menten equation.

To study the structure and function of the SegC protein, we expressed and isolated it from *E. coli* BL21 DE3. First, we employed a fluorescence polarization (FP) DNA-binding assay and electrophoretic mobility shift assay (EMSA) to investigate a potential DNA-binding ability of SegC. Our results show that SegC displays weak DNA-binding activity with no specific sequence preference, with a dissociation constant (*K*_d_) of ∼10^−6^ μM (Figure 1A and B and Supplementary Figure S2). The DNA-binding affinity of SegC is similar to that of SegA (∼1.3 μM), but it is 12-fold lower than that observed for SegB (∼0.18 μM) (18). The weak DNA-binding affinity suggests that the association of SegC with DNA might be rather transient. Despite attempts to study the solution state of SegC by gel filtration, interactions between the protein and the gel filtration matrix impeded this analysis. Even when we used a high-salt buffer (1.8M NaCl), we were unable to observe SegC protein (Supplementary Figure S3A). Since the Superdex 200 (Cytiva) matrix contains cross-linked agarose and dextran, SegC may interact with glycocyclic substrates in the gel filtration matrix. To further confirm this hypothesis, we used 6 M guanidine chloride (GdnHCl) to elute SegC. As shown in Figure S3B, we can detect SegC protein through SDS-PAGE, confirming that SegC sticks to the gel filtration matrix under non-denaturing conditions. Since we were unable to determine the native state of SegC via gel filtration, we used a BS^3^ cross-linking assay to investigate its potential multimeric states in solution. Our results revealed that SegC can exist as monomer, dimer, and higher oligomeric states (Supplementary Figure S4). The potential to oligomerize may be connected with the function of SegC.

### SegC forms multimeric structures

**Figure 2.**
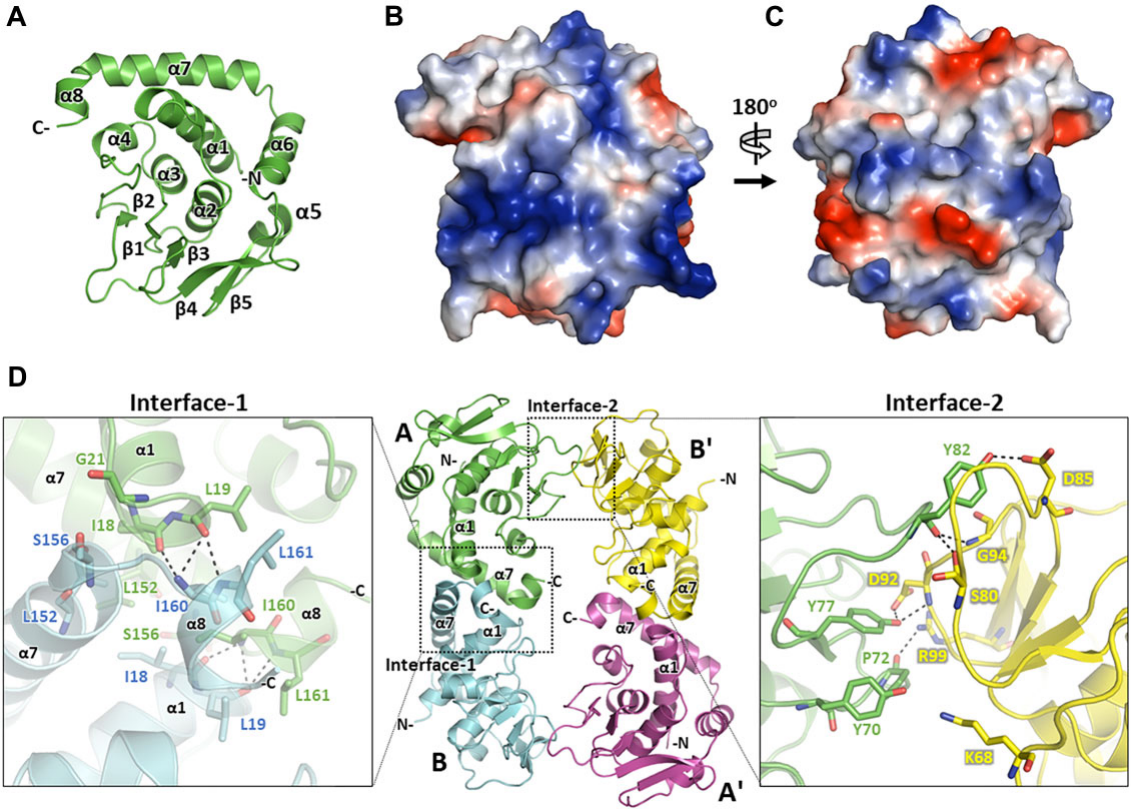
Structures of the SegC monomeric and tetrameric conformations. (**A**) The SegC monomer is shown as a ribbon, and its eight α-helixes (α1–α8) and five β-strands (β1–β5) have been labeled. (**B**) Electrostatic surface potentials of the SegC monomer. (**C**) Rotated view (180° along the y-axis of the structure in Figure 2B) of SegC monomer. Positively- and negatively-charged residues are colored in blue and red, respectively. (**D**) The SegC tetramer. The four molecules (labeled A, B, A' and B') are shown in green, cyan, magenta, and yellow, respectively. Zoomed-in views of Interface-1 or Interface-2 are shown on the right or left-hand side of the SegC tetramer, respectively. The dotted line squares indicate SegC dimer Interface-1 or Interface-2. Zoomed-in representations of Interface-1 and Interface-2 are shown at right and left, respectively. The residues involved in the interaction are shown as sticks and have been labeled.

Since the function of SegC is elusive, we attempted to use structural information to elucidate its potential activities. To this end, we determined the crystal structure of SegC at 2.8 Å resolution. The monomer structure of SegC exhibits a compact spherical shape consisting of eight α-helices and a five-stranded parallel β-sheet (Figure 2A). The α-helices and β-sheet cluster on either side of the SegC molecule. Two long loops (α4β2 loop and β2β3 loop) are located on the same side as part of the β-sheet. In that vicinity, residues Cys88 and Cys91 form a disulfide bond between the β3 and β3β4 loop (Supplementary Figure S5). In addition, the electrostatic surface potential on one side of SegC reveals two positively-charged grooves (Figure 2B), which might play a key role in the DNA-binding ability of SegC. In contrast, the opposite side of SegC bears a more mixed charge distribution (Figure 2C).

In the asymmetric unit, SegC forms a tetramer composed of two types of dimers, i.e. AB and AB' (Figure 2D). All four monomers exhibit an almost identical conformation, with the root mean square deviation (r.m.s.d.) ranging from 0.20 to 0.25 Å (in Cα). To determine the most likely interaction interface that could represent a major structural element in tetramer formation, we used the PISA server (30) to measure the surface areas of these two dimer interfaces. The buried surfaces within dimer AB and dimer AB' are 713.4 and 497.4 Å^2^, respectively (Interface-1 and Interface-2 of Figure 2D, respectively). In addition, PISA calculations show that AB dimer can be stably assembled. Therefore, Interface-1 of dimer AB most likely plays a major role in the formation of the SegC dimer, and the interaction between the two dimers contributes to the assembly and stabilization of the entire tetramer.

The main forces involved in Interface-1 and Interface-2 are hydrogen bonds and hydrophobic interactions (detailed interactions are shown in Figure 2D). The major residues that contribute to Interface-I are Ile160 and Leu161 from the C-terminal α8 helix of SegC. In addition, the α8 helix of molecule A and the α1 helix of molecule B form a helix dipole interaction. Residues of the α1 and α7 helixes, as well as the loop region between α7 and α8 contribute hydrophobic interactions. Furthermore, interactions in the SegC AB' dimer interface (Interface-2) are primarily attributable to loop-rich regions, comprising the α4β2, β2β3 and β3β4 loops of both monomers. Moreover, all residues involved in dimer Interface-1 (Ile18, Leu19, Leu152, Ser156, Ile160 and Leu161) are conserved in the genera *Saccharolobus* and *Sulfolobus*. There are 10 amino acids involved in SegC dimer Interface-2 (Lys68, Try70, Pro72, Tyr77, Ser80, Tyr82, Asp85, Asp92, Gly94 and Arg99), but only four of those residues (Lys68, Tyr77, Gly94 and Arg99) are conserved in both *Saccharolobus* and *Sulfolobus* (Supplementary Figure S1). We also noticed that two SegC tetramers can assemble into an octamer from two adjacent asymmetric units (Supplementary Figure S6A).

During the crystallization trials, we also observed another crystal form under different condition after a couple of months of crystal screening. After further structural determination, we were surprised to find that only monomer structure was present in the asymmetric unit, hereafter referred to as SegC_m_ (Supplementary Figure S6B). The r.m.s.d. between the SegC_m_ and SegC monomers is 0.42 Å (in Cα), implying that the overall structure does not change much. However, the electron density in the C-terminal region of SegC_m_ (residues 155–165) is missing (Supplementary Figure S6C and D). The loss of the C-terminal region of SegC_m_ (residues 155–165) may result in an inability to form the AB dimer interface, thereby disrupting dimer formation (Supplementary Figure S6C and D). To confirm that the missing C-terminus was not due to a flexibility issue, we dissolved SegC_m_ crystals and measured their molecular weight by mass spectrometry. The results of mass spectrometry showed two main peaks for the SegC_m_ crystal of 18038.6 Da and 18143.8 Da, which were significantly smaller than the molecular weight peak for SegC (19859.7 Da) (Supplementary Figure S6E). This difference in molecular weight corresponds to a peptide of approximately fourteen residues (including six his-tag), implying that residues after Gly159 are degraded during crystallization. Thus, the extreme C-terminal region of SegC is crucial for dimer formation, as its degradation destroys the AB dimer interface, preventing dimer assembly and even further affecting the genesis of multimers. Therefore, we constructed the deletion mutant SegC_1–155_ (residues 1–155) to further elucidate the function of SegC.

### Docking model of SegC interacting to DNA

Although we tried to obtain the SegC-DNA complex, acquiring structural information remained challenging and further efforts are still needed. To provide further insights into how SegC binds to non-specific DNA, we used the HDOCK online server (http://hdock.phys.hust.edu.cn/) to predict possible docking models of SegC tetramer with dsDNA (31). The top five structures with HDOCK scores share two sets of DNA binding modes. Moreover, the two sets of docking models show symmetrical similarities. The DNA model of the AB molecule is symmetrically oriented with the DNA model of A'B'. Therefore, the structure with the highest HDOCK score was selected as the best modeling structure of the SegC-DNA complex for further analysis. As shown in Supplementary Figure S7, the docking model shows that DNA is located on a surface rich in positively charged residues. These interactions are dominated by electrostatic interactions on molecule A', while molecule B' provides only few interactions (Supplementary Figure S7A and B). The SegC residues that may interact with DNA are, in order, Lys46, Lys68, Arg73, Arg81, Asn90 and Arg99 (Supplementary Figures S7C and D). In addition, we performed a DNA docking analysis using SegC dimer to verify its DNA binding patterns. As shown in Supplementary Figure S7E and F, most of the SegC dimer–DNA docking models presented similar binding modes to the SegC tetramer-DNA docking models, and they shared the same DNA-binding residues (Supplementary Figure S7). Moreover, our docking model indicates that S1 dsDNA potentially packs within SegC filamentous structures (Supplementary Figures S7).

To determine the role of those SegC residues involved in DNA binding, we constructed corresponding variants including K46A, K68A, R73A, R81A, N90A and R99A through site-directed mutagenesis. Regrettably, K68A cannot be overexpressed. We measured the DNA-binding ability of these mutant proteins by fluorescence polarization (FP). As shown in Supplementary Figure S8A, most of the SegC mutant proteins have similar DNA-binding affinities to that of wild-type (Figure 1A). Only the binding affinity of SegC-N90A to DNA was two-fold lower than that of WT, with a *K*_d_ value of approximately 4.58 μM (Supplementary Figure S8A). Given that we have already shown that SegC is a non-sequence-specific DNA-binding protein (Figure 1B), like bacterial ParA proteins SegC may rely on multiple positively-charged residues to associate with DNA (32,33). In such a case, if a single positively-charged residue is changed, there may be little or no effect on DNA binding. Instead, multiple residues need to be mutated to observe a substantial reduction in DNA-binding affinity. Therefore, we designed double and triple mutations at different positions based on Asn90, including K46A/N90A, R81A/N90A and K46A/R81A/N90A. However, none of these SegC double and triple mutants could be overexpressed, so we were unable to elucidate the DNA-binding ability of SegC through these mutants. To further understand whether the affinity of SegC for DNA is mediated by electrostatic interactions, we performed DNA binding assays at a salt concentration of 500 mM. As expected, SegC lost its DNA-binding ability under high-salt conditions (Supplementary Figure S8B). These findings suggest that SegC binds to DNA through electrostatic interactions. In addition, single mutations can only affect the binding affinity of SegC for DNA, but cannot completely destroy the DNA-binding ability of SegC.

### SegC structure reveals unique substrate-binding features

To explore links between the structure and function of SegC, we performed a structural similarity search with the Dali server (34). According to our search results (Supplementary Table S2), SegC shows the highest structural homology to the thiamine diphosphate-binding (ThDP-binding) fold superfamily of proteins (such as branched-chain α-keto acid decarboxylase/dehydrogenase (E1b), pyruvate dehydrogenase (E1p), and transketolase). However, SegC only shares a low sequence identity of 5% to 15% in the corresponding regions with similar structure. Supplementary Figure S9 shows the results of the structural comparison between SegC and E1b (PDB ID: 1V1M) by the Dali server. The superimposition between SegC and E1b has the r.m.s.d of 3.0 Å (in Cα), aligned by 151 residues out of a total of 165 residues of SegC (Supplementary Figure S10 and Supplementary Table S2). Based on the structural comparison, the α-helix and β-sheet regions of SegC have similar tertiary structure folding to those of the ThDP-binding fold superfamily (Supplementary Figure S9 and Supplementary Figure S10). However, SegC has no obvious sequence similarity with those proteins (Supplementary Figure S10).

As shown in Supplementary Figure S9B, the ThDP-binding residues of E1b are located in the loop region between the α-helix and β-sheet. However, the corresponding area in SegC is a loop-rich region. This structural difference may indicate that SegC binds to different substrates. Furthermore, the ThDP molecule has two phosphate groups, and the residues responsible for binding these phosphates in E1b are Arg114, Glu193 and Arg220. Surprisingly, the corresponding residues in SegC are Lys46, Asn90 and Arg99, respectively. These residues are also located in the strip-like positively charged region and, consequently, they are likely to be important for nucleotide binding. To further explore the ThDP-binding-like motif in SegC, we performed a BLAST search in the Thiamine diphosphate (ThDP)-dependent Enzyme Engineering Database (TEED) (35), but did not get any hits. This outcome indicates that SegC is not a ThDP-binding protein. However, since the structure of SegC has a similar fold to that of ThDP-binding proteins, SegC may bind substrates that harbor phosphate groups or ring-containing molecules.

### SegC displays NTP hydrolyzing activity

**Figure 3.**
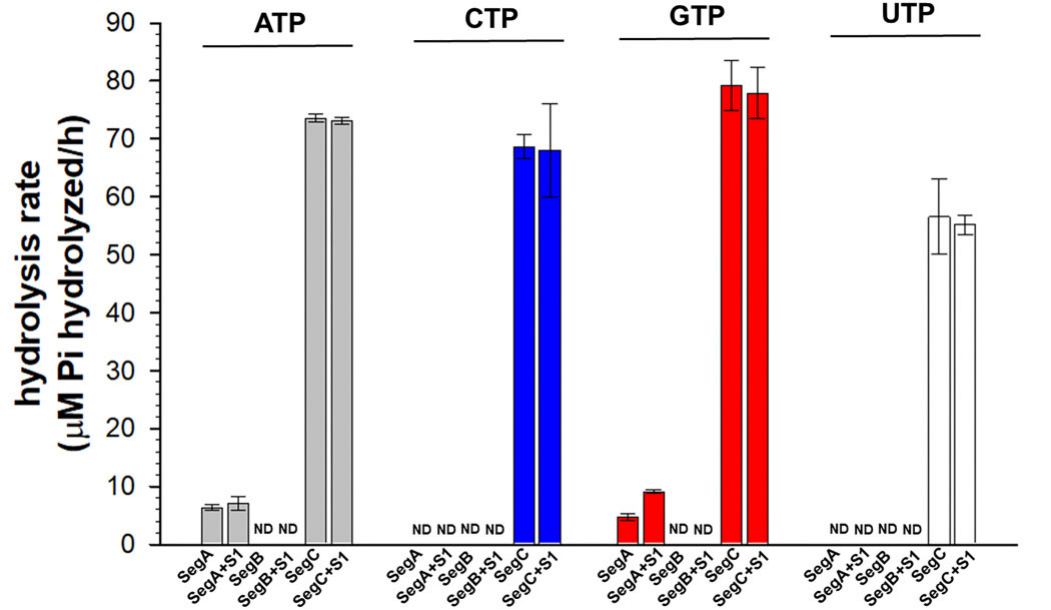
SegC hydrolyzes NTPs. ATP, CTP, GTP and UTP are colored gray, blue, red and white, respectively. SegA and SegB proteins were used as positive and negative controls, respectively. S1: 21-bp site 1 dsDNA. ND: not detected. All measurements were performed in triplicate, and error bars represent the standard deviation of the mean.

The superfamily of ThDP-binding motifs forms a large and diverse group of proteins with varying substrate specificities and catalytic activities, though most substrates are ring-containing compounds such as thiamin diphosphate (36,37). Interestingly, recent studies have shown that the ParB protein in bacterial ParABS chromosome segregation systems can bind and hydrolyze CTP to CDP (38–40). We wondered if SegC may also bind to nucleotides and potentially catalyze their hydrolysis. To test this hypothesis, we determined if SegC can hydrolyze NTPs (ATP/CTP/GTP/UTP) by measuring inorganic phosphate accumulation. To our surprise, we observed that SegC exhibited catalytic activity against all of the tested NTPs with no apparent preference, although its activity was slightly lower against UTP (Figure 3). For all of the tested NTPs, 10 μM SegC hydrolyzed approximately 70 μM NTP per hour (Figure 3). We also used 10 μM SegA and SegB as positive and negative controls to confirm that SegC does indeed possess NTPase activity. As shown in Figure 3, SegA displayed weak ATPase activity, only hydrolyzing ∼10 μM ATP per hour, which is consistent with previous studies (17,18). Interestingly, we also observed that SegA showed low GTP hydrolytic activity of ∼5 μM GTP per hour (Figure 3). Notably, several chaperone proteins, such as Hsp60 and Hsp90, exhibit ATPase and GTPase activities (41,42). Therefore, proteins with ATP/GTPase activity are not uncommon. As expected, SegB displayed no catalytic activity against all tested NTPs (Figure 3). Earlier study also showed that the ParB_Bsu_ dimers only hydrolyzed about five CTP molecules per hour (38). In fact, ParA and SegA (the bacterial and archaeal chromosome segregating members) are mentioned to have only above basal ATPase activity (17,18,33). These studies indicated that most chromosome segregation proteins in bacteria and archaea have weak ATPase or CTPase activities. Thus, we think that SegC indeed has NTPase activity. Moreover, previous studies have shown that the ATPase/CTPase activities of components of chromosome segregation systems, such as ParA and ParB, are enhanced in the presence of DNA (33,38). However, we found that DNA does not affect the NTPase activity of SegC (Figure 3; SegC + S1).

Although our results indicate that SegC has broad-specific NTP hydrolytic activity, structural information about the NTP-binding site in SegC is still lacking. To explore potential NTP binding sites, we attempted to co-crystallize SegC with ATP without success. Therefore, we performed an ATP soaking experiment. After several trials, we determined the crystal structure by soaking apo-form crystal in crystallization solution with additional 20 mM ATP. From *F*_o_ – *F*_c_ ligand omit electron density map, we observed an additional electron density in the interface of the two dimers under the ATP-soaked condition (Supplementary Figure S11A), which was not observed in apo-form density map (Supplementary Figure S11B). Based on the shape and size of the electron density, we can only fit an ADP molecule. This indicated that the ATP molecule has been hydrolyzed to ADP. In this SegC-ADP complex structure, two residues (Tyr61 and Arg73) were found to interact with ADP. Residue Arg73 forms hydrogen-bond interactions with two phosphate groups, and residue Tyr61 is involved in a π–π stacking interaction with adenine. Since the ADP is positioned at the interface of the two dimers, we also identified two residues, Arg22 and Asp25, from different molecules. However, these two residues are located more than 4 Å away from ADP (Supplementary Figure S11A).

We then constructed two mutant proteins, SegC-Y61A and SegC-R73A, and examined their NTPase activities. The results showed that the NTPase activity of the SegC-Y61A mutant was increased 49% relative to wild-type SegC (SegC-WT), whereas that of SegC-R73A mutant was decreased by 35% compared to SegC-WT (Supplementary Figure S11C). Based on our structure, residue Tyr61 stabilizes the adenine moiety of ADP (Supplementary Figure S11A). Therefore, mutation of the Tyr61 residue to alanine disrupts the interaction with adenine, causing ADP to be easily released, potentially explaining why the SegC-Y61A mutant has higher NTPase activity. This phenomenon has also been reported previously for bacterial ATP-binding cassette (ABC) transporters that interact with the adenine ring of bound ATP by an aromatic residue, with mutation of the aromatic amino acid resulting in enhanced ATP release (43). In terms of SegC residue Arg73, its side chain has a hydrogen bond with the phosphate group of ADP, showing that this residue is one of the NTPase active site residues (Supplementary Figure S11A). Together, these results potentially indicate that SegC is a non-canonical NTPase. In addition, the SegC-ADP complex has a different catalytic site compared to that of E1b-ThDP (Supplementary Figure S9E and F), and both Tyr61 and Arg73 are involved in SegC NTP hydrolysis activity, but neither is located in the ThDP catalysis site. However, we cannot rule out the involvement of additional SegC residues in NTP hydrolysis processes.

### SegC and DNA form filaments in the presence of NTPs

**Figure 4.**
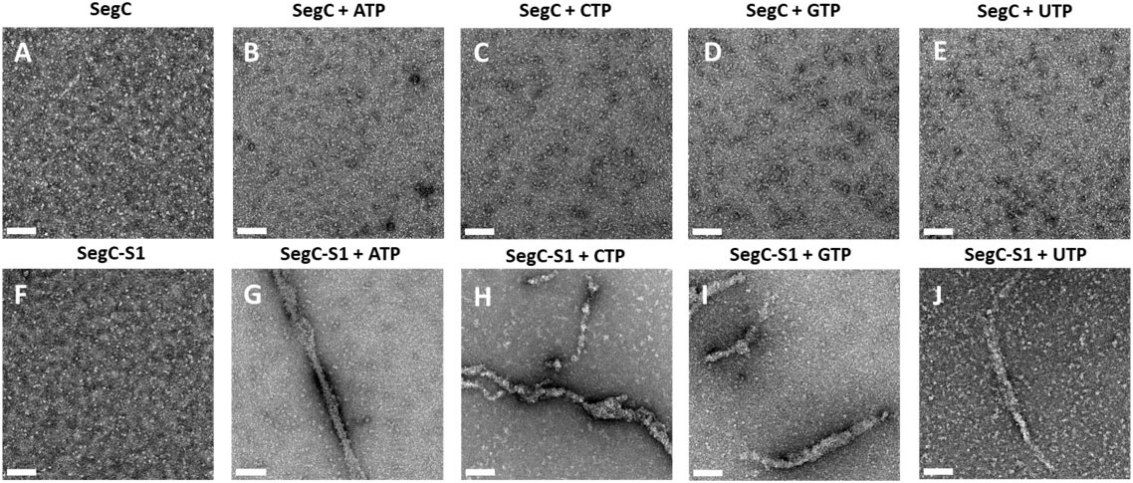
Negative-stain electron microscopy images of SegC in the presence or absence of DNA and NTP. (**A**) SegC only. (**B**) SegC + ATP. (**C**) SegC + CTP. (**D**) SegC + GTP. (**E**) SegC + GTP. (**F**) SegC-S1. (**G**) SegC-S1 + ATP. (**H**) SegC-S1 + CTP. (**I**) SegC-S1 + GTP. (**J**) SegC-S1 + UTP. S1: 23-bp site 1 dsDNA. Scale bar = 100 nm.

To further explore the effect of NTPs on SegC, we used negative-stain electron microscopy (EM) to capture the structure of SegC with or without the S1 DNA site in the presence of different NTPs (Figure 4). The resulting images revealed small and irregular particles, either for SegC alone or in the presence of the 23-bp S1 DNA (Figure 4A and F). We also observed a similar pattern for SegC in the presence of NTPs (Figure 4B–E). Interestingly, SegC formed filaments, when both NTPs and the S1 DNA were present (Figure 4G–I). These filaments ranged in width from 15.2 to 25.1 nm and varied in length. The filamentous structures were observed when any of the ribonucleoside triphosphates was added to the reaction. Thus, NTPs can assist SegC to assemble into filaments in the presence of DNA *in vitro*.

### SegA induces higher-order filament formation

**Figure 5.**
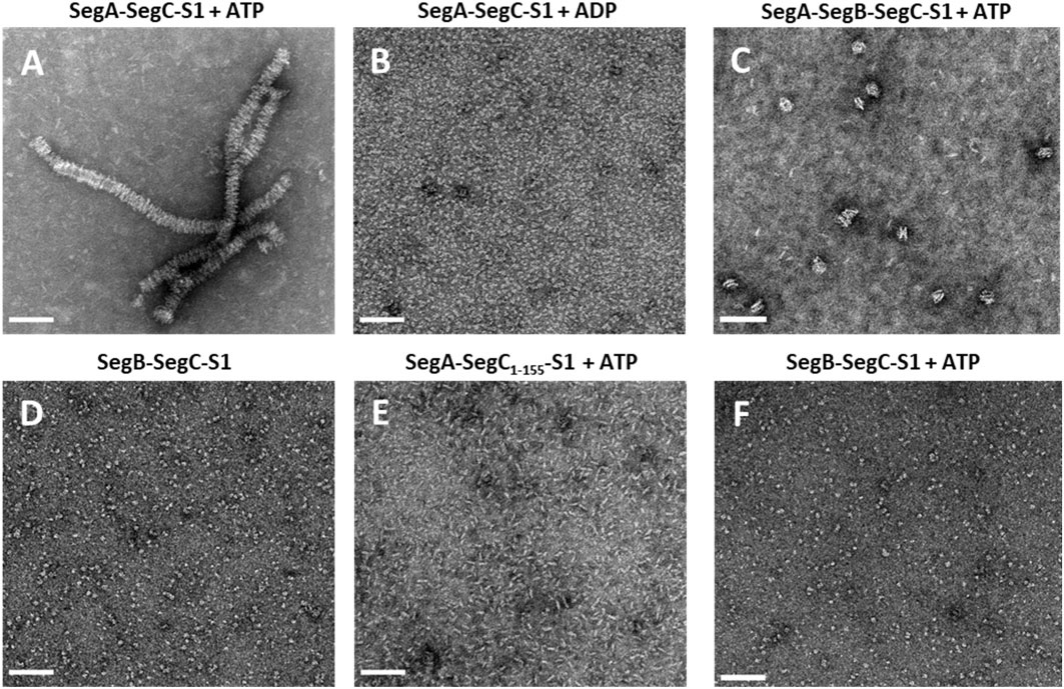
Cooperation between SegC, SegA, SegB, and DNA, as revealed by negative-stain electron microscopy. (**A**) SegA-SegC-S1 + ATP. (**B**) SegA-SegC-S1 + ADP. (**C**) SegA-SegB-SegC-S1 + ATP. (**D**) SegB-SegC-S1. (**E**) SegA-SegC_1–155_-S1 + ATP. (**F**) SegB-SegC-S1 + ATP. S1: 23-bp site 1 dsDNA. Scale bar = 100 nm.

To further explore if SegC functions in archaeal chromosome segregation, we again deployed negative-stain EM to capture the structure of SegC in reactions containing various combinations of SegA, SegB and DNA (Figure 5). Although we have already established that SegC does not display any catalytic preference for ATP, CTP, GTP or UTP, physiological concentrations of intracellular ATP are relatively higher than those of the other NTPs (44). Accordingly, for subsequent experiments, we focused on the interactions of these components (SegA, SegB, SegC and S1 DNA) with ATP.

**Figure 6.**
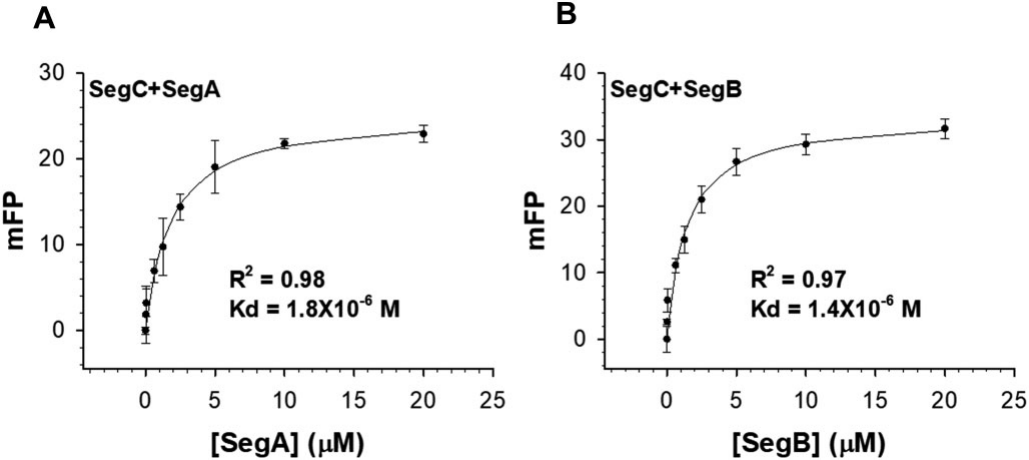
SegC associates with SegA and SegB. Fluorescence polarization studies performed on (**A**) SegA or (**B**) SegB with fluoresceinated SegC. All measurements are reported in triplicate and error bars represent the standard deviation of the mean. The solid lines represent fitting curves to the Michaelis–Menten equation.

First, SegC was co-incubated with SegA and the 23-bp S1 DNA in the presence of ATP, which resulted in a large number of uniform and long filaments (Figure 5A). These assemblies appeared to be more textured structure than the SegC-NTPs filaments (Figure 4) and appear to consist of seemingly repeated units. To validate our observations, we conducted the same experiment with ADP instead of ATP. As shown in Figure 5B, the large and long filaments were no longer observed in the presence of ADP, indicating that filament formation is ATP-dependent. Furthermore, these large filament structures were disrupted upon addition of the SegB protein (Figure 5C), potentially because SegB significantly enhances the ability of SegA to hydrolyze ATP (18). To gain further insights into the function of SegC, we incubated SegB with SegC in the presence of the 23-bp S1 DNA and observed many small-sized particles (Figure 5D). However, we did not detect the SegB-S1 helical partitioning complex observed in our previous study (18), indicating that SegC may interact with SegB, thereby perturbing SegB's DNA-binding properties. The SegA-SegC-S1 + ATP reaction showed long filaments (Figure 5A), so we further examined this combination by replacing SegA with SegB to investigate the role of ATP in SegB, SegC and S1 DNA interactions. As shown in Figure 5F, the particles of SegB-SegC-S1 + ATP looked similar to those of SegB-SegC-S1 (Figure 5D). This result indicates that ATP does not affect particle formation by the SegB, SegC and DNA mixture (Figure 5D). Next, we wanted to investigate whether SegC has direct contact with SegA and/or SegB. To this end, we used fluorescence polarization binding assay to determine whether SegC interacts with SegA and SegB. The results showed that SegC indeed interacts with SegA and SegB with binding affinities of 1.8 and 1.4 μM, respectively (Figure 6). This finding suggests that SegC interacts directly with both SegA and SegB and filament dynamics are likely regulated through those interactions.

### The SegC C-terminus is important for maintaining protein structure and function

Our crystal structure revealed that the C-terminus of SegC (residues 156–165) is the main interface for dimer formation (Figure 2D). To confirm the importance of the C-terminus, we constructed a mutant protein (SegC_1–155_) in which the last 10 residues (156–165) were deleted. First, we performed a fluorescence polarization DNA-binding assay to examine the binding affinity of SegC_1–155_ for DNA, which showed that SegC_1–155_ lacked DNA-binding ability (Figure 1C). Next, we assessed if SegC_1–155_ can still form filament. To do so, we applied negative stain EM with different combinations of SegC_1–155_, ATP and S1 DNA. As shown in Supplementary Figure S12, all resulting images presented similar small particles, with no filaments being formed even in the presence of three components. Thus, SegC dimers represent the basic unit for forming tetramers or higher oligomers to assemble into filaments. In addition, we also examined the filament formation ability of SegC_1–155_ with SegA in the presence of both S1 DNA and ATP. As shown in Figure 5E, we did not observe long and uniform filaments. Thus, truncation of the SegC C-terminal region may disrupt the protein's ability to form dimer, thereby impairing its function. However, we cannot exclude the possibility that the SegC C-terminal region (residues 156–165) may be involved in the SegC–SegA interaction.

## Discussion

Previous studies on Sulfolobales have revealed that chromosome replication is followed by a prolonged G2 phase, during which the nucleoid changes shape and undergoes highly organized compaction (14–16). The two replicated chromosomes are later aligned in the cell before segregation (14–16), which then occurs rapidly during M phase (14,15). In bacteria, chromosome replication and segregation occur simultaneously (45), which implies fundamental regulatory differences between these two kingdoms of life. Overall, Crenarchaeota exhibit a cell cycle similar to that of eukaryotes.

In this study, we focused on a protein of unknown function, SegC. Surprisingly, we found that SegC forms filament in the presence of DNA and NTPs (Figure 4G–J). Notably, presence of SegA further remodels these filaments into having a larger diameter (∼40 nm) in the presence of ATP (Figure 5A). In addition, SegB can modulate the properties of these filaments, likely by promoting the ATPase activity of SegA (Figure 5C). The formation of filaments by SegC in the presence of SegA, DNA and ATP, as well as regulation of their dynamics by SegB, indicates that SegC may play a role in archaeal chromosome segregation. Further studies are needed to understand whether this filamentation property of SegC affects or involves the whole chromosome or is limited to specific regions. However, the dynamics we observed *in vitro* indicate that the assembly and disassembly of SegC filaments are regulated by SegA and SegB, which indeed supports that SegC is associated with the *S. solfataricus* chromosome segregation complex. The filamentation behaviour of SegC may play a structural/architectural role in the chromosome segregation process, although, at this stage, we cannot exclude the possibility that SegC might exert a regulatory function in the dynamics of SegAB complex formation and lifespan in the cell.

These observations are reminiscent of the bacterial ParMR*C* filament-pushing system (46–48) (Supplementary Figure S13A). In the ParMR*C* system, ParM forms dynamic, actin-like filaments that segregate plasmids in a mitosis-like process (49). In the presence of ATP, ParM assembles into short filaments that can undergo catastrophic disassembly upon ATP hydrolysis (49). However, the ParR-*parC* complex, other components of the ParMR*C* system, act as a cap to stabilize bipolar elongation. Furthermore, ParM cannot form filaments alone, necessitating both ParR and *parC* (49). Although SegC is not a ParM ortholog, it may adopt a similar mechanism to mediate filament formation.

The filamentation behaviour of chromosome segregation systems is believed to provide the mechanism by which the chromosome movement is facilitated (49). In the bacterial ParAB*S*and ParMR*C* systems, DNA movement is mainly attributable to ATP hydrolysis by ParA and ParM proteins (Supplementary Figure S13). According to the sequence identity, SegA is a ortholog of ParA, indicating that SegA is involved in chromosome segregation. (17,18). In the bacterial ParA, DNA dissociation and association are regulated by ATP hydrolysis and is a crucial mechanism for mediating DNA movement (Supplementary Figure S13B). However, the SegA DNA binding ability is not restricted by ATP (18). Therefore, how SegA promotes chromosome segregation is an interesting question. Here, our study found that another partner protein, SegC, forms filament in the presence of DNA and ATP. Interestingly, the filaments formed by SegC were reorganized when SegA was present, but were disassembled upon addition of SegB (Figure 5), thus highlighting a potential mechanism of DNA separation.

Based on the observation that SegA and SegB proteins are produced in early S phase (19,20), chromosomes may begin to be organized by these proteins during DNA replication (14). SegB may mediate DNA compaction around specific sites in synergy with SegA. As SegA binds DNA non-specifically, it may be stochastically patterned throughout the chromosome. At a later stage of the cell cycle, once compaction has been completed, SegC may engage with SegA and DNA in the ATP state, and the proteins may assemble into filaments that nucleate at the site where SegA is bound to DNA. These filaments may facilitate separation of sister chromosomes. Towards the conclusion of this process, SegB may mediate dissolution of the SegAC filaments.

**Figure 7.**
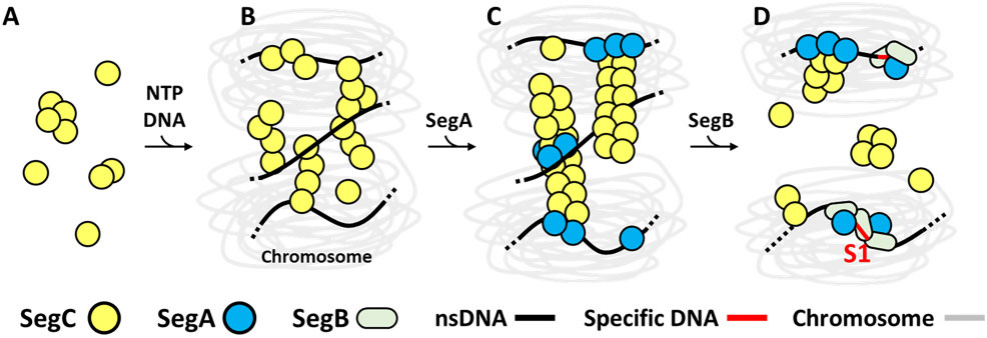
A hypothetical model for SegC filament function. The proposed mechanism comprises four steps. (**A**) SegC is randomly distributed in the archaeal cell, where it may form multimers. (**B**) Upon binding to NTP and DNA, SegC forms filaments. (**C**) In the presence of SegA, the SegC-DNA + NTP filaments are remodeled into larger, higher-order filaments. (**D**) Presence of SegB stimulates SegA ATPase activity, resulting in filament dissociation (18).

According to previous findings and the results presented herein, we propose a speculative model for how SegC, SegA and SegB might cooperate in chromosome segregation (Figure 7). Initially, irregular filaments form when NTP-bound SegC randomly associates with chromosomal DNA. Thereafter, incorporation of SegA triggers remodeling of the SegC-DNA filaments, generating higher-order filaments, that promote chromosome segregation. Then, participation of SegB stimulates the ATPase activity of SegA, prompting filament disassembly. Although different aspects of this model remain to be further elucidated and corroborated by *in vivo* investigations, our study indicates that SegC interacts with the archaeal SegAB chromosome segregation system and that SegC likely plays a role in the separation of the chromosomes prior to cell division. Future investigations will provide further insights into the enticing roles played by the SegC protein.

## Supplementary Material

gkae660_Supplemental_File

## Data Availability

All the data supporting the findings of this study are available within the paper, and Supplementary files. The atomic coordinates and structure factors of SegC, SegC_m_ and SegC-ADP have been deposited in the Protein Data Bank (PDB) with ID codes 8WQ8, 8WQN and 8YK9, respectively.
