# Supplementary material for: Unraveling the structure and function of a novel SegC protein interacting with the SegAB chromosome segregation complex in Archaea
Source: Nucleic Acids Res. 2024 Jul 30;52(16):9966–77. doi: 10.1093/nar/gkae660 (PMC11381335; doi:10.1093/nar/gkae660)
Supplement: gkae660_Supplemental_File [file gkae660_supplemental_file.pdf]

Supplementary Information

Unraveling the structure and function of a novel SegC protein interacting with the SegAB chromosome segregation complex in Archaea

Min-Guan Lin[1,†], Cheng-Yi Yen[1,†], Yo-You Shen[1], Yu-Sung Huang[2], Irene W Ng[3], Daniela Barillà[3], Yuh-Ju Sun[2,*], Chwan-Deng Hsiao[1,*]

[1] Institute of Molecular Biology, Academia Sinica, Taipei 115, Taiwan

[2] Institute of Bioinformatics and Structural Biology, National Tsing Hua University, Hsinchu 300, Taiwan

[3] Department of Biology, University of York, Wentworth Way, York YO10 5DD, United Kingdom

[†]These authors contributed equally to this work

***For correspondence:** yjsun@life.nthu.edu.tw, hsiao@gate.sinica.edu.tw

This PDF file includes:

Supplementary Figures S1 to S13

Supplementary Tables S1 to S2

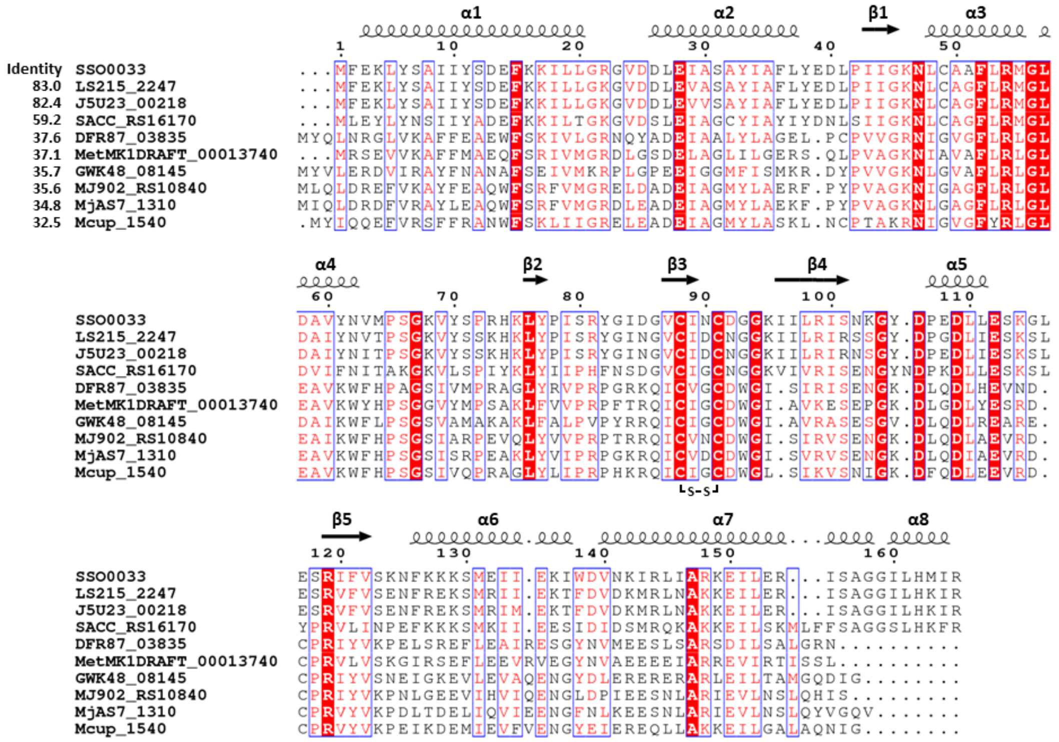

Supplementary Figure S1. Multiple sequence alignments of SegC. Multiple sequence alignment of the SegC and blast result with different *Sulfolobaceae*. *Saccharolobus solfataricus* SegC (SSO0033), *Saccharolobus shibatae* LS215_2247 (LS215_2247), *Sulfolobus islandicus* J5U23_00218 (J5U23_00218), *Saccharolobus caldissimus* SACC_RS16170 (SACC_RS16170), *Metallosphaera hakonensis* DFR87_03835 (DFR87_03835), *Metallosphaera Yellowstonensis* MetMK1DRAFT_00013740 (MetMK1DRAFT_00013740), *Metallosphaera tengchongensis* GWK48_08145 (GWK48_08145), *Metallosphaera sedula* MJ902_RS10840 (MJ902_RS10840), *Metallosphaera javensis* MjAS7_1310 (MjAS7_1310), and *Metallosphaera cuprina* Mcup_1540 (Mcup_1540) are shown. The disulfide bond of SegC is labelled. All sequence alignments were drawn and analyzed by Clustal Omega and ESPript3 (1)

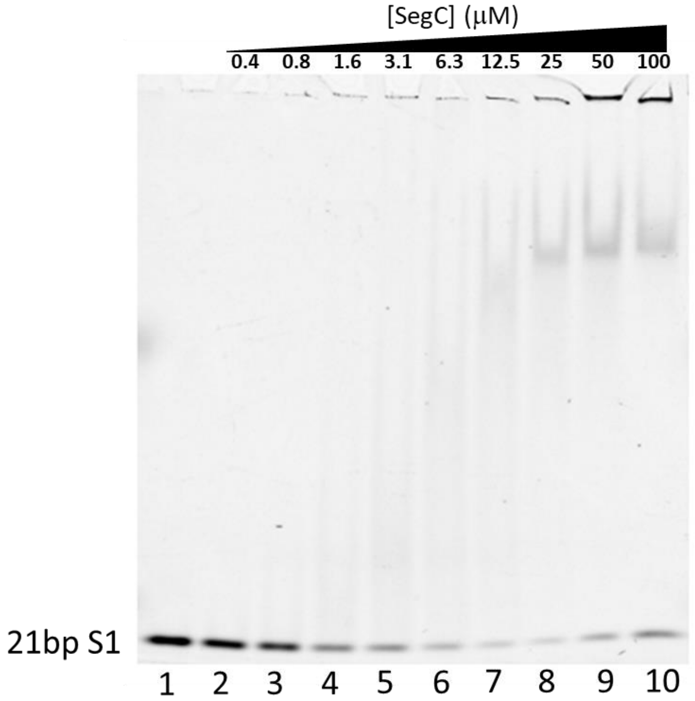

Supplementary Figure S2. DNA binding ability of SegC. Representative EMSA in which increasing concentrations (0.4-100 μM) of SegC were incubated with 10 nM 5' end Cy3-labeled 21bp S1 dsDNA. Lanes 1 to 10 at the following concentrations: 0, 0.4, 0.8, 1.6, 3.1, 6.3, 12.5, 25, 50 and 100 μM. The reactions were run on 4-12% TBE gel.

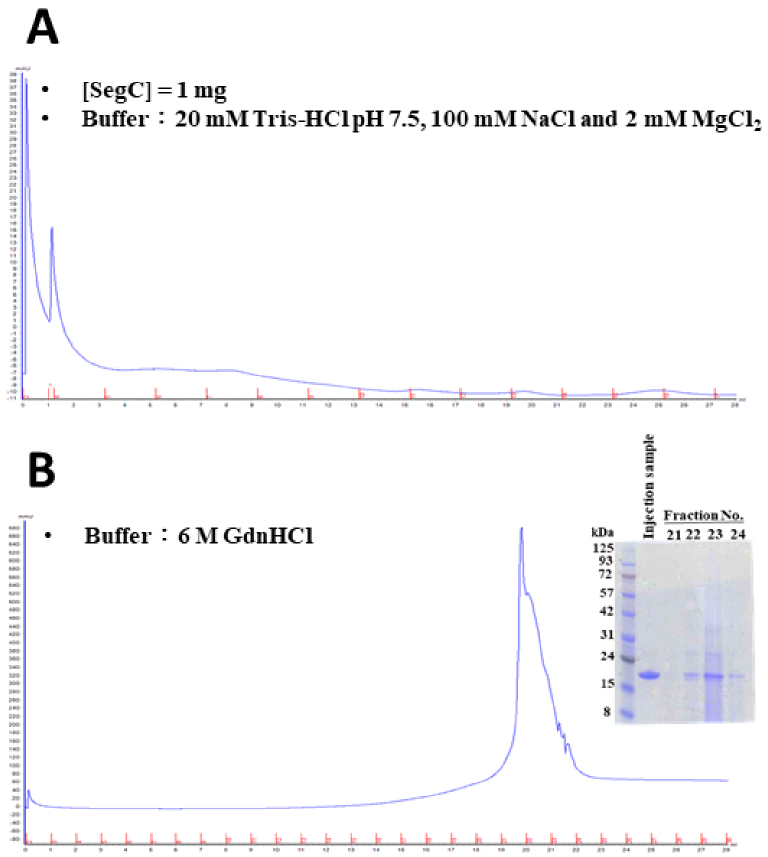

Supplementary Figure S3. Size exclusion chromatography result for SegC. (**A**) Gel filtration profiles of SegC. (B) Gel filtration profile of SegC with denaturant. SDS-PAGE analysis of injection sample and elution peak fraction. The maker (M) size was shown in kDa.

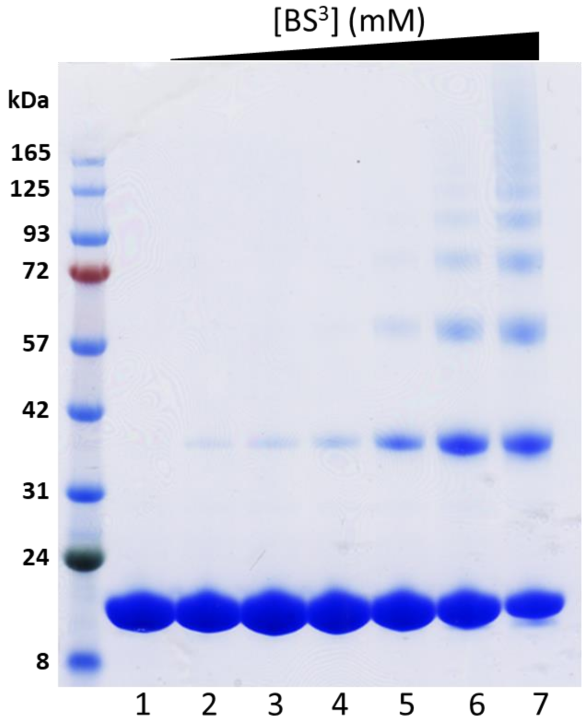

Supplementary Figure S4. Oligomerization state of SegC. Cross-linking with crosslinker BS^3. SegC was cross-linked using BS^3 in order to assess the oligomerization state. Lanes 1 to 7 correspond to 30 min of incubation with BS^3 at the following concentrations: 0, 0.125, 0.25, 0.5, 1, 2 and 4 mM. Cross-linking products were analyzed in 4-12% SDS-PAGE gels.

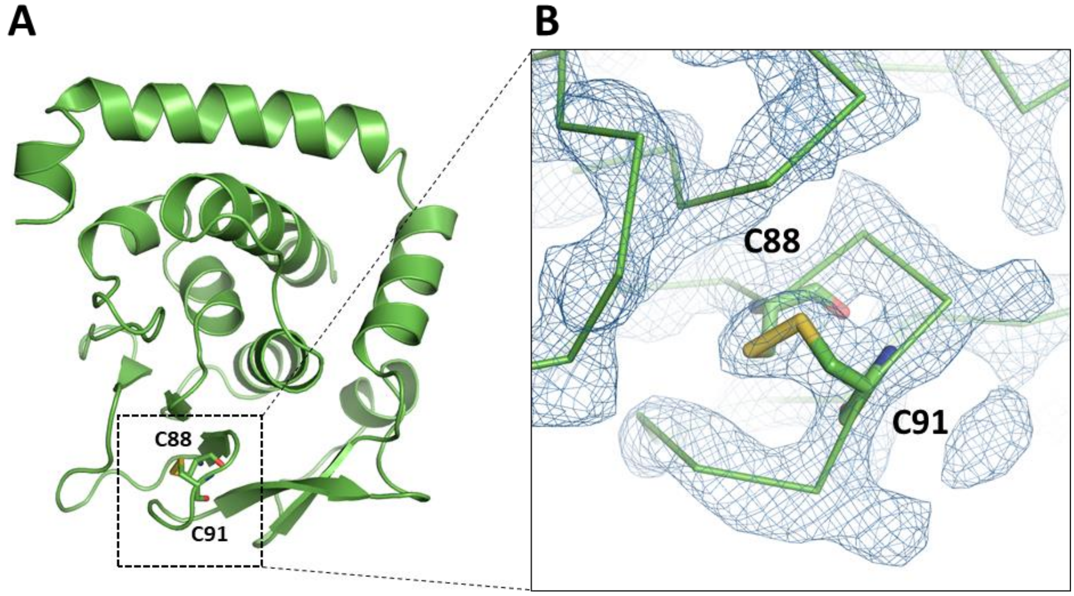

Supplementary Figure S5. Disulfide bond of SegC structure and its omit electron density map. (**A**) The disulfide bond between Cys88 and Cys91 is shown as sticks and labeled as dotted cycle. (**B**) The F_o - F_c omit electron density maps of disulfide bond is contoured at 3.0 σ and shown as a mesh.

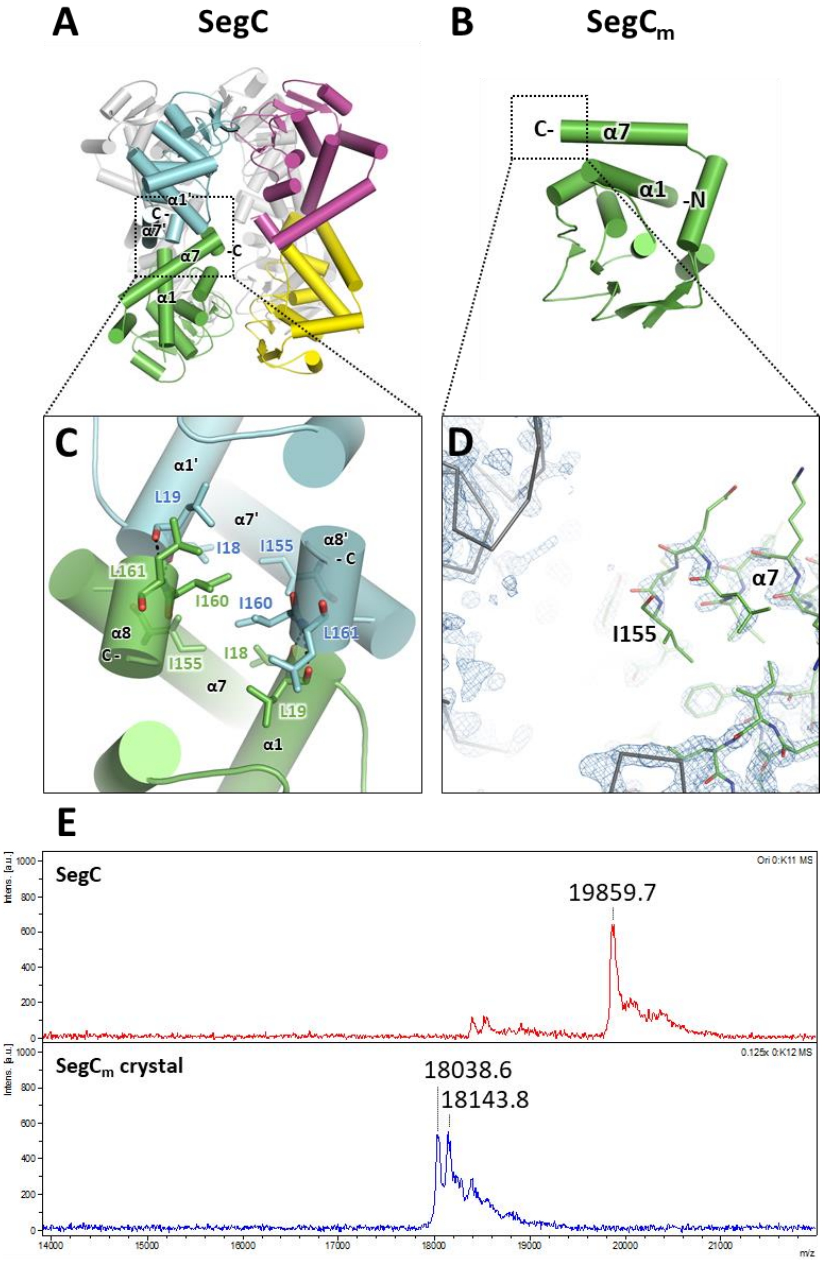

Supplementary Figure S6. Assemblies of SegC structure and mass spectrometry of $SegC_m$. (**A**) Two asymmetric units of the SegC structure are displayed as ribbon. Each SegC molecule is colored in green, cyan, magenta, or yellow, respectively. The neighboring asymmetric unit is colored in gray. (**B**) The asymmetric unit of $SegC_m$ structure is shown as a ribbon model. (**C**) A close-up dimer interface view of SegC structures from Supplementary Figure 4A is presented. (**D**) The F_o - F_c omit electron density maps of $SegC_m$ helix α7 is contoured at 3.0 σ and shown as a mesh. (**E**) The mass spectrum of SegC and $SegC_m$ crystal is shown at the upper and down panels, respectively.

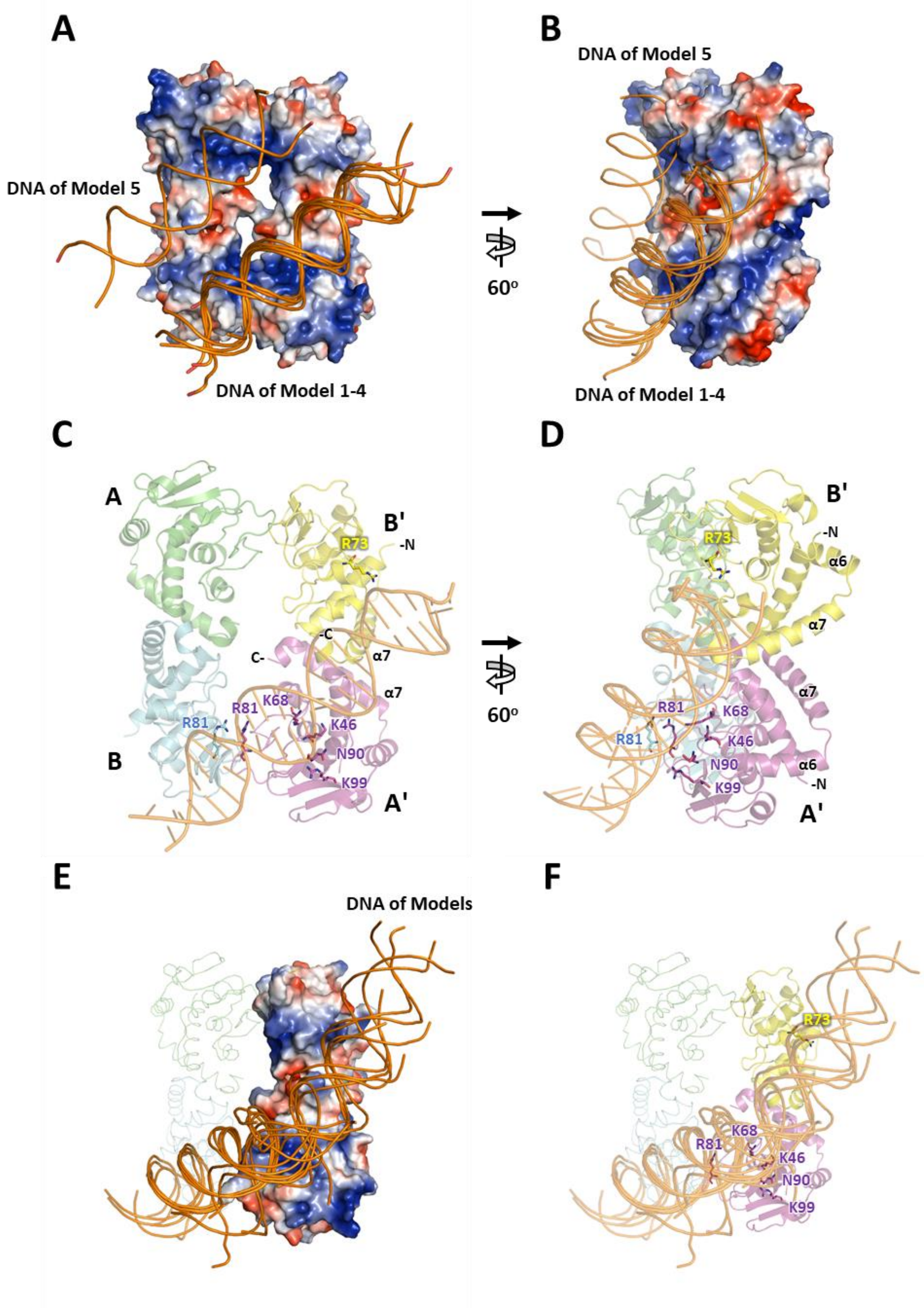

Supplementary Figure S7. Molecular docking of SegC with DNA using HDOCK. (A) The top five docking score structures of the SegC-DNA complex model were performed using the HDOCK online server. SegC displays electrostatic surface potentials, while DNA is represented as a ribbon model. Positively- and negatively-charged residues are colored in blue and red, respectively. (B) A rotated view (60° along the y-axis of the structure in Figure S6A) of the SegC-DNA complex structural models is presented. (C) Detailed potential electrostatic or H-bond interaction residues between SegC and DNA within 4 Å are displayed. The four molecules (labeled A, B, A', and B') correspond to

the molecules in Figure 2D, shown in green, cyan, magenta, and yellow, respectively. (D) Rotated view (60° along the y-axis of the structure in Figure S6C) of SegC-DNA complex structural model. The random 24 bp dsDNA from PDB structure (6IUB) was used for the docking model. (E) The DNA docking models of the SegC dimer. The SegC dimer displays electrostatic surface potentials, whereas DNA is represented as a ribbon and the corresponding tetramer position is represented as a thinner ribbon. (F) Detailed potential electrostatic or H-bond interaction residues between SegC dimer and DNA within 4 Å are displayed. The random 24 bp dsDNA from PDB structure (6IUB) was used for the docking model.

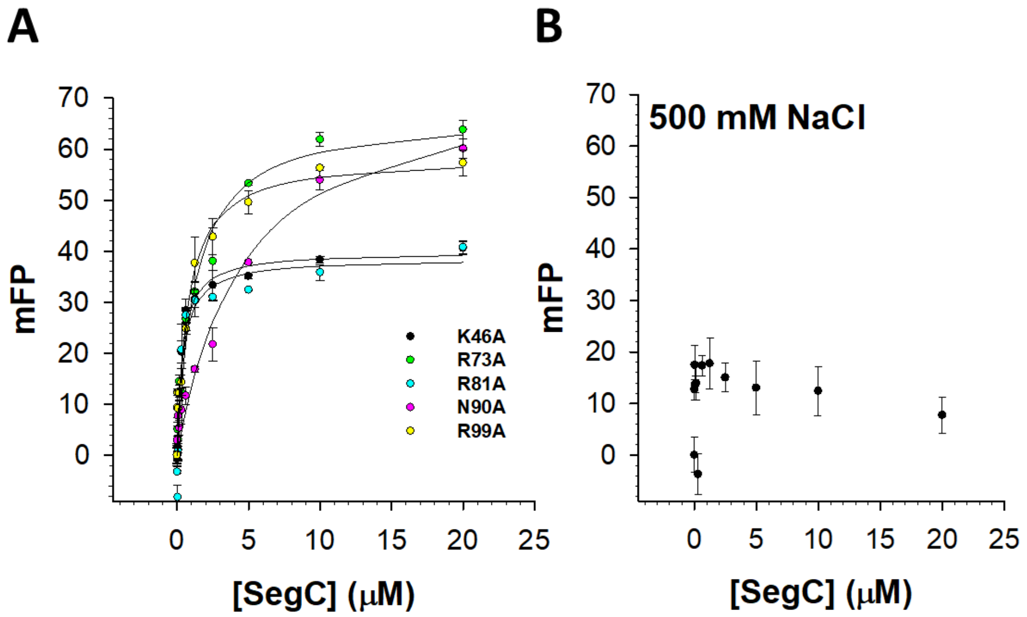

SegC mutant	Kd value
SegC-K46A	0.37 μM (R^2 = 0.98)
SegC-R73A	1.24 μM (R^2 = 0.98)
SegC-R81A	0.40 μM (R^2 = 0.96)
SegC-N90A	4.58 μM (R^2 = 0.97)
SegC-R99A	0.74 μM (R^2 = 0.96)

Supplementary Figure S8. The equilibrium DNA binding assays of SegC and its mutant proteins with S1 DNA were done by fluorescence polarization binding isotherms (FP). T. (A) The S1 DNA binding for SegC-K46A (blue), SegC-R73A (red), SegC-R81A (cyan), SegC-N90A (magenta), SegC-R99A (yellow) were shown. The corresponding Kd are shown in the table below. (B) The effect of high salt concentration in SegC DNA binding ability. The Kd is NB (no measurable binding). DNA binding was measured by fluorescence polarization (in mFP units), plotted against protein concentration (0–20 μM). The average of three independent experiments is shown with error bars representing one standard deviation of the mean.

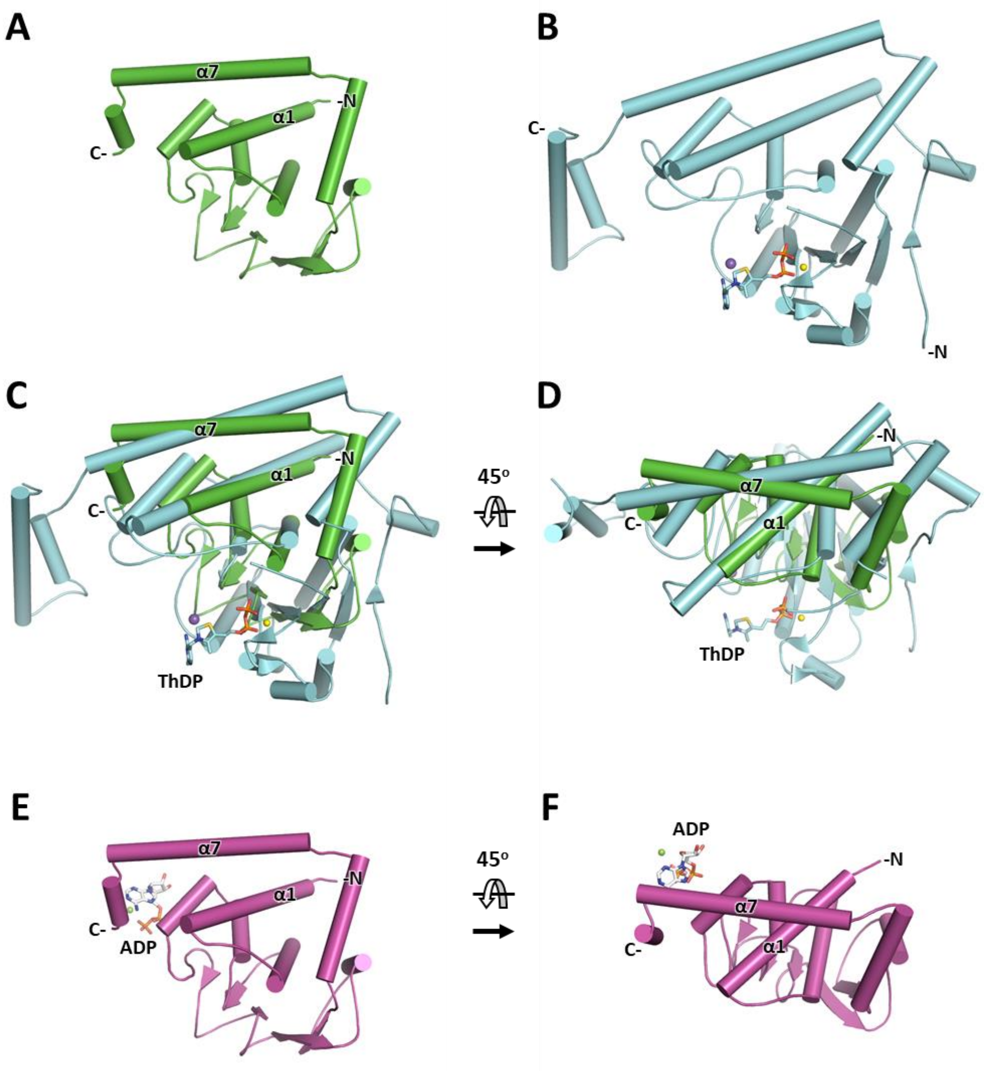

Supplementary Figure S9. Structural comparison of SegC and E1b. (**A**) SegC and (**B**) branched-chain α-keto acid decarboxylase/dehydrogenase (E1b) (PDB ID:1V1M) structure are displayed as ribbon. The thiamine diphosphate (ThDP) is shown in a stick. Manganese and potassium ions are shown and colored as yellow and purple spheres, respectively. (**C**) Structural superimposition of SegC and E1b is according to the analyzed result of Dali. (**D**) SegC and E1b structures are rotated 45°. (**E**) The SegC-ADP structure shows the same orientation as the SegC and E1b structure comparison. (**F**) SegC-ADP structures are rotated 45°. ADP is shown as a stick, and the magnesium ion is shown as a green sphere.

```
DSSP         --------------------------------------------------LLHHHHHHHH
SegC         --------------------------------------------------MFEKLYSAII 10
Identity                                                          |||
E1b (1V1M)   kpqfpgasaefidklefiqpnvisgipiyrvmdrqgqiinpsedphlpkeKVLKLYKSMT 60
DSSP         llllllllllllllllllllllllllleellllllllllllhhhlllllhhHHHHHHHHHH

               α1                            α2           β1         α3
DSSP         HHHHHHHHHHL----------LLLLHHHHHHHHHHHHL-LLLLEEE--LLHHHHHHHHHh
SegC         YSDEFKKILLG----------RGVDDLEIASAYIAFLY-EDLPIIG--KNLCAAFLRMGl 57
Identity              ||                   |      |          |          |
E1b (1V1M)   LLNTMDRILYEsqrqgrisfyMTNYGEEGTHVGSAAALdNTDLVFGqyREAGVLMYRDY- 119
DSSP         HHHHHHHHHHHhhhlllllllLLLLLLHHHHHHHHHHLlLLLEEELllLLHHHHHHLLL-

               α4                               β2
DSSP         hHHHHL----------------lLLLLlLLHHHLEELL------LLLLLL----------
SegC         dAVYNV----------------mPSGKvYSPRHKLYPI------SRYGID---------- 85
Identity                            |             |
E1b (1V1M)   -PLELFmaqcygnisdlgkgrqmPVHY-GCKERHFVTIssplatQIPQAVgaayaakran 177
DSSP         -LHHHHhhhhlllllllllllllLLLL-LLLLLLLLLLllllllHHHHHHhhhhhhhhhl

                   β3                               β4
DSSP         ----LEEELL-------------------lllLEEEEEE---------------------
SegC         ----GVCINC-------------------dggKIILRIS--------------------- 101
Identity           |                          |
E1b (1V1M)   anrvVICYFGegaasegdahagfnfaatlecpIIFFCRNngyaistptseqyrgdgiaar 237
DSSP         llllEEEEEEllhhhlhhhhhhhhhhhhhlllEEEEEEElleelleehhhlllllllhhh

                               α5                    β5          α6
DSSP         -------eEEEL---HHHHHHL--LLLL--------LEEEELLL-hhHHHHHHHHHHL--
SegC         -------nKGYD---PEDLLES--KGLE--------SRIFVSKN-fkKKSMEIIEKIW-- 138
Identity                |                          |
E1b (1V1M)   gpgygimsIRVDgndVFAVYNAtkEARRravaenqpFLIEAMTYrigDHPISRLRHYLls 297
DSSP         hhhhlleeEEEElllHHHHHHHhhHHHHhhhhhlllEEEEEELLlllLLHHHHHHHHHhh

                              α7           α8
DSSP         -------LHHHHHHHHHHHHHHHHHHhLHHH-----------------------------
SegC         -------DVNKIRLIARKEILERISAgGILH----------------------------- 162
Identity                 |   |    |
E1b (1V1M)   qgwwdeeQEKAWRKQSRRKVMEAFEQ-AERKpkpnpnllfsdvyqempaqlrkqqeslar 356
DSSP         hllllhhHHHHHHHHHHHHHHHHHHH-HHHLllllhhhhlllllllllhhhhhhhhhhhh


DSSP         -------------hhl
SegC         -------------mir 165
Identity
E1b (1V1M)   hlqtygehypldhfdk 372
DSSP         hhhhhhhhllhhhlll
```

Supplementary Figure S10. Pairwise structural alignments of SegC and E1b by the Dali severer. Three-state secondary structure definitions by DSSP (reduced to H=helix, E=sheet, L=coil) are shown above the amino acid sequence. Structurally equivalent residues are in uppercase, structurally non-equivalent residues (e.g. in loops) are in lowercase. Amino acid identities are marked by vertical bars. DSSP is an acronym for Dictionary of Secondary Structure of Proteins. The two 3/10 (η) helices (η1 = G56-N62; η2 = P72-H74) are define as H.

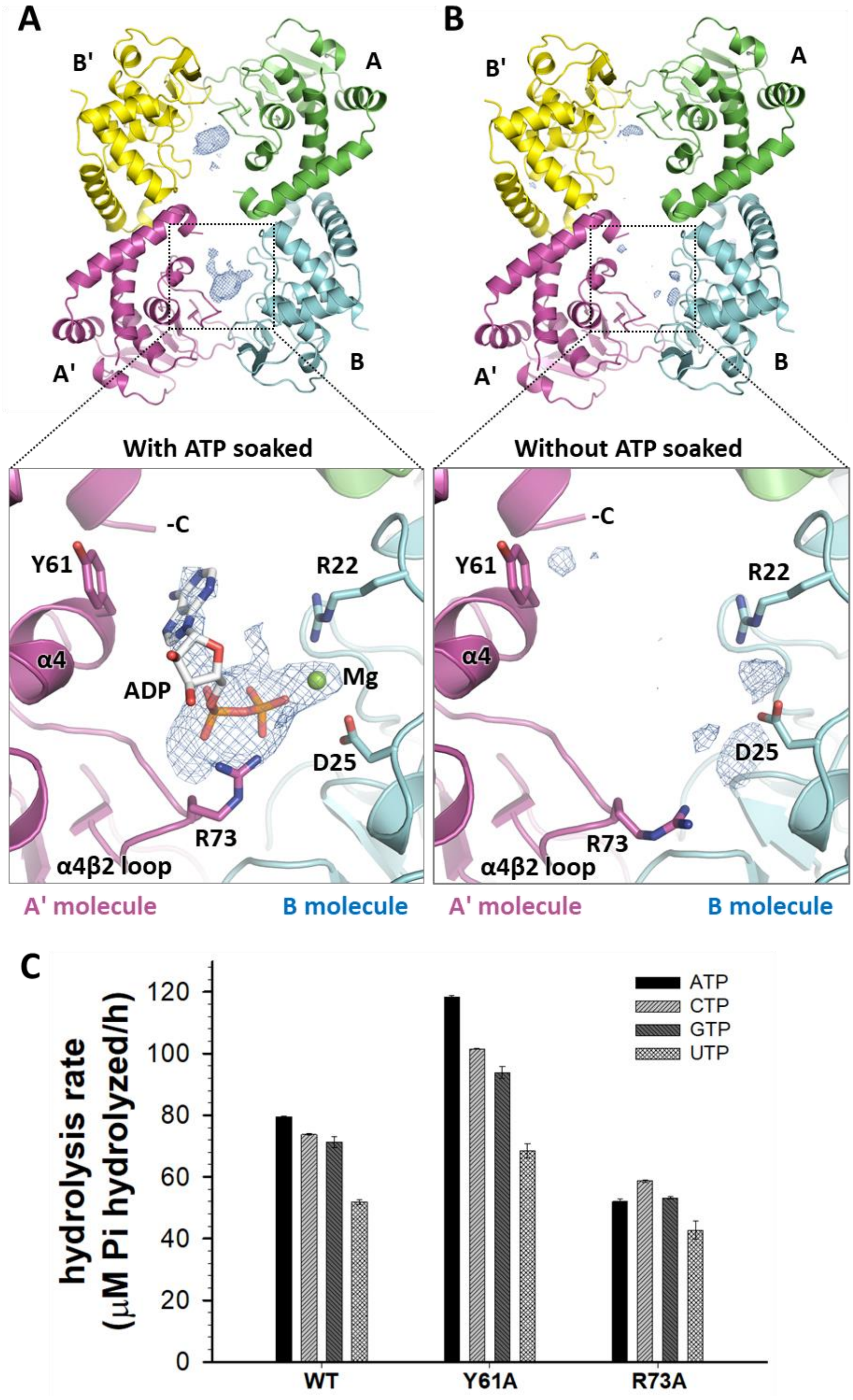

Supplementary Figure S11. The ligand omit map of SegC structure without or with ATP soaked. (A) The ligand omit map of SegC structure with ATP soaked. ADP is shown as a stick, and the magnesium ion is shown as a green sphere. The F_o - F_c ligand omit electron density maps of ADP, and magnesium is contoured at 3.0 σ and shown as a mesh. Omit maps were generated without the ADP and magnesium from the structure.

The molecules (labeled A' and B) correspond to the molecules in Figure 2D, shown in magenta and cyan, respectively. (B) The ligand omit map of SegC structure without ATP soaked. (C) The NTPase activity of SegC and its mutant. To assess SegC WT and its mutant to NTP hydrolyze activity in the different nucleotide (ATP, CTP, GTP and UTP). All measurements were performed in triplicate, and error bars represent the standard deviation of the mean.

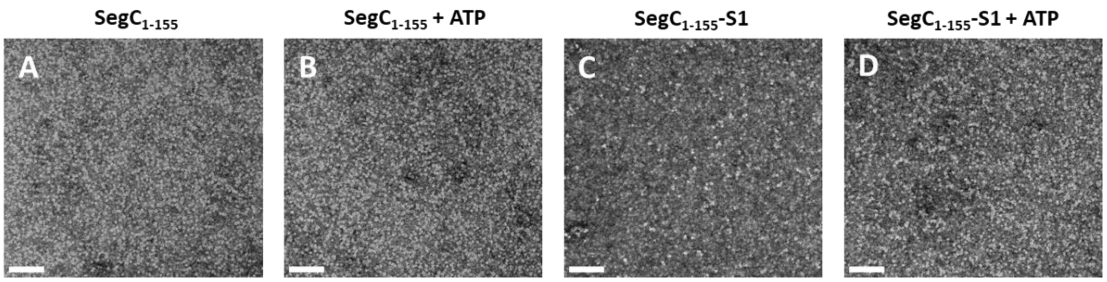

Supplementary Figure S12. Negative-stain electron microscopy images of $SegC_{1-155}$ in the presence or absence of DNA and ATP. (A) $SegC_{1-155}$ only. (B) $SegC_{1-155}$ + ATP. (C) $SegC_{1-155}$-S1. (D) $SegC_{1-155}$-S1 + ATP. S1: 23-bp site 1 dsDNA. Scale bar = 100 nm.

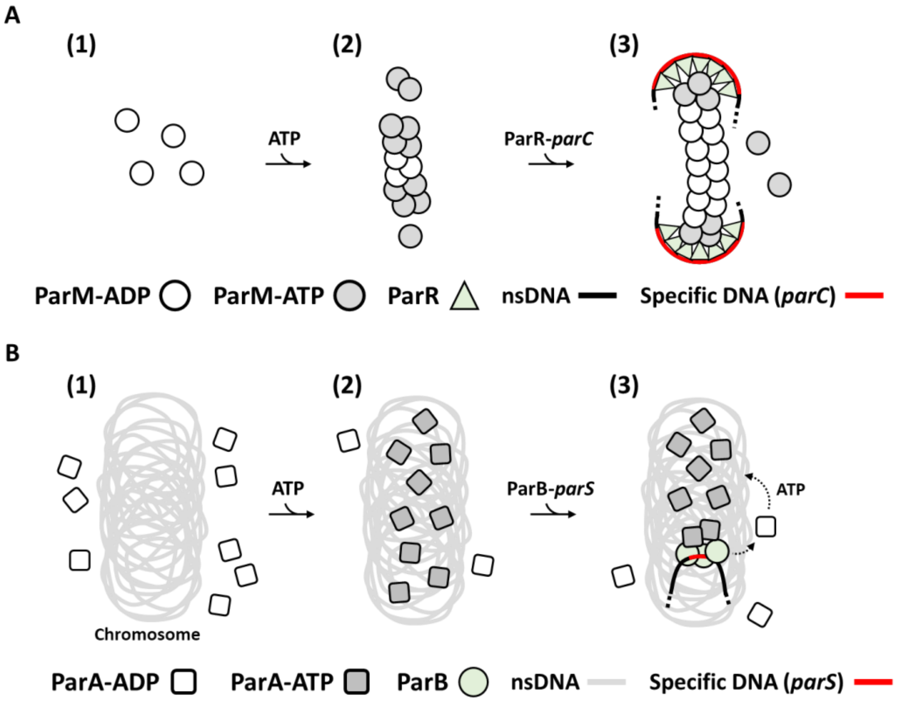

Supplementary Figure S13. Schematic diagram of bacterial ParMRC and ParABS molecular mechanism. (A) The molecular model of the ParMRC system. (1) ParM is randomly distributed in the cell. (2) ParM forms short filaments in the presence of ATP, which are unstable and prone to disassembly beyond the limit length. (3) After the ParR-*parC* complex caps the ParM filament, the ParM undergo a stable elongation. The ParR-*parC* complex is then pushed and moved by the elongated ParM filament (pushing model). (B) The diffusion-ratchet model of the ParABS system. (1) ParA is randomly distributed in the cell. (2) ParA non-specifically binds to the nucleoid in the presence of ATP. (3) After nucleoid-bound ParA interacts with the ParB-*parS* complex, ParA hydrolyzes ATP, losing its DNA binding ability and releasing from the nucleoid. ParA then exchanges ADP for ATP and binds to the nucleoid again. At this time, the ParB-*parS* complex is attracted and moves to another nucleoid-bound ParA. The result of these cycles of binding and ATP hydrolysis is a net movement of the *parS*-proximal oriC region of the chromosome towards the opposite pole of the cell (diffusion ratchet model).

Supplementary Table S1. X-ray diffraction data and refinement statistics of SegC.

Crystal	SegC	$SegC_m$	SegC-ADP
**Data collection statistics**			
Source	NSRRC-TPS 07A	NSRRC-TPS 05A	NSRRC-TPS 05A
Wavelength (Å)	0.97625	0.99984	1.00003
Space group	$P4_32_12$	$P2_12_12_1$	$P4_32_12$
Resolution (Å)	2.8	1.8	3.4
Unit cell parameters			
a, *b*, *c* (Å)	156.9 / 156.9 / 127.0	33.9 / 70.2 / 71.8	159.2 / 159.2 / 127.9
Redundancy of reflection	6.4 (6.6)[a]	4.1 (4.1)	6.3 (6.2)
Completeness (%), overall	99.9 (100.0)	96.3 (98.8)	99.8 (99.9)
$I/\sigma(I)$, overall	16.9 (2.6)	17.1 (3.4)	17.6 (2.4)
R_{merge}[b] (%), overall	10.3 (76.7)	7.2 (38.7)	7.8 (55.0)
$CC_{1/2}$, overall	0.988 (0.785)	0.992 (0.866)	0.986 (0.829)
**Refinement statistics**			
Resolution (Å)	29.4 - 2.8	28.1 - 1.8	29.7 - 3.4
R-factor[c]/R_{free}[d] (%)	18.2 / 21.5	18.6 / 22.0	19.7 / 22.9
Number of reflections used	39,461	15,757	22,971
Number of residues	660	155	660
Number of atoms			
Protein	5,280	1,248	5,280
Water	176	127	-
ADP	-	-	108
Mg	-	-	1
B-factor ($Å^2$)			
Protein	64.1	22.3	96.5
Water	62.2	36.1	-
ADP	-	-	153.9
Mg	-	-	94.95
RMSD bond lengths (Å)	0.009	0.007	0.012
RMSD bond angles (°)	0.982	0.869	1.381
PDB ID[e]	8WQ8	8WQN	8YK9

[a] Values in parentheses are for the highest-resolution shell.
[b] $R_{merge}=\Sigma|I-\langle I\rangle|/\Sigma I$, where *I* is the observed intensity and $\langle I\rangle$ is the average intensity from multiple observations of symmetry-related reflections.
[c] $R=\Sigma|F_{obs}-F_{calc}|/\Sigma F_{obs}$, where F_{obs} and F_{calc} are the observed and calculated structure factor amplitudes, respectively.
[d] R_{free} was calculated with 5% of the total number of reflections randomly omitted from the refinement.
[e] Protein data bank identifiers for co-ordinates.

Supplementary Table S2. The structural homologs protein of SegC identified by Dali and showing the top twenty results of PDB90.

	PDB ID	Z-score[a]	r.m.s.d. (Å)	Lali	Nres	Identity (%)	Description	Organism
**1**	1V1M	10.0	3.0	151	372	12	2-oxoisovalerate dehydrogenase alpha subunit	Homo sapiens
**2**	2BFF	9.5	3.0	152	392	13	2-oxoisovalerate dehydrogenase alpha subunit	Homo sapiens
**3**	1QS0	9.0	3.0	150	407	9	2-oxoisovalerate dehydrogenase alpha subunit	Pseudomonas putida
**4**	1UM9	8.8	3.1	146	335	11	2-oxo acid dehydrogenase alpha subunit	Thermus thermophilus
**5**	1UMB	8.3	3.0	145	362	10	2-oxo acid dehydrogenase alpha subunit	Thermus thermophilus
**6**	3EXE	7.7	3.2	147	363	10	pyruvate dehydrogenase E1 component subunit alpha	Homo sapiens
**7**	3EXG	7.6	3.2	146	342	12	pyruvate dehydrogenase E1 component subunit alpha	Homo sapiens
**8**	3DV0	7.4	3.2	141	344	13	pyruvate dehydrogenase E1 component subunit	Geobacillus stearothermophilus
**9**	1W85	7.3	3.4	141	365	15	pyruvate dehydrogenase E1 component, alpha subunit	Geobacillus stearothermophilus
**10**	2JGD	6.2	3.6	141	811	9	2-oxoglutarate dehydrogenase E1 component	Escherichia coli (strain K12)
**11**	2XT6	5.8	3.5	147	1055	7	2-oxoglutarate decarboxylase	Mycolicibacterium smegmatis MC2 155
**12**	2Y0P	5.6	3.3	143	856	6	2-oxoglutarate decarboxylase	Mycolicibacterium smegmatis MC2 155
**13**	6U3J	5.6	3.3	147	876	13	2-oxoglutarate dehydrogenase E1 component DHKTD1	Homo sapiens
**14**	8P5V	5.4	3.4	141	1121	8	2-oxoglutarate dehydrogenase E1/E2 component	Corynebacterium glutamicum ATCC 13032
**15**	7WGR	5.1	3.4	138	850	9	2-oxoglutarate dehydrogenase, mitochondrial	Homo sapiens
**16**	6KM9	4.5	3.4	132	845	8	Oxoglutarate dehydrogenase (Succinyl-transferring), E1 component	Vibrio vulnificus CMCP6
**17**	8P5S	4.5	3.5	137	1087	8	2-oxoglutarate dehydrogenase E1/E2 component	Corynebacterium glutamicum ATCC 13032
**18**	6VEF	4.2	3.4	137	843	9	2-oxoglutarate dehydrogenase E1 component	Escherichia coli K-12
**19**	1R9J	4.0	3.6	119	671	8	transketolase	Leishmania mexicana mexicana
**20**	6RJB	3.6	3.7	110	622	8	transketolase	Homo sapiens

PDB90: PDB structures with less than 90% sequence identity to each other.
Z-score: statistical significance of the similarity between SegC and structurally related proteins.
r.m.s.d: root mean square deviation of aligned Cα atoms.
Lali: number of align structurally equivalent residues in Cα.
Nres: number of residues in target structure.
Identity (%): percent identity of aligned structurally equivalent residues.
